# Mitochondrial dysfunction is a key determinant of the rare disease lymphangioleiomyomatosis and provides a novel therapeutic target

**DOI:** 10.1038/s41388-018-0625-1

**Published:** 2018-12-20

**Authors:** E. M. M. Abdelwahab, S. Pal, K. Kvell, V. Sarosi, P. Bai, R. Rue, V. Krymskaya, D. McPhail, A. Porter, J. E. Pongracz

**Affiliations:** 1 Department of Pharmaceutical Biotechnology, School of Pharmacy, University of Pecs, Pecs, Hungary; 2 Janos Szentagothai Research Centre, University of Pecs, Pecs, Hungary; 3 Department of Pharmaceutical Technology, School of Pharmacy, University of Pecs, Pecs, Hungary; 4 Department of Internal Medicine, School of Medicine and Clinical Centre, University of Pecs, Pecs, Hungary; 5 Department of Medical Chemistry, MTA-DE Lendulet Laboratory of Cellular Metabolism, University of Debrecen, Debrecen, Hungary; 6 Research Center for Molecular Medicine, Faculty of Medicine, University of Debrecen, Debrecen, Hungary; 7 Pulmonary, Allergy and Critical Care Division, Department of Medicine, Perelman School of Medicine, University of Pennsylvania, Philadelphia, PA USA; 8 Antoxis Ltd, Aberdeen, UK; 9 School of Medicine and Medical Sciences, University of Aberdeen, Aberdeen, UK

**Keywords:** Target identification, Autophagy

## Abstract

Lymphangioleiomyomatosis (LAM) is a rare and progressive systemic disease affecting mainly young women of childbearing age. A deterioration in lung function is driven by neoplastic growth of atypical smooth muscle-like LAM cells in the pulmonary interstitial space that leads to cystic lung destruction and spontaneous pneumothoraces. Therapeutic options for preventing disease progression are limited and often end with lung transplantation temporarily delaying an inevitable decline. To identify new therapeutic strategies for this crippling orphan disease, we have performed array based and metabolic molecular analysis on patient-derived cell lines. Our results point to the conclusion that mitochondrial biogenesis and mitochondrial dysfunction in LAM cells provide a novel target for treatment.

## Introduction

Ten-year survival of the progressive and systemic orphan disease, Lymphangioleiomyomatosis (LAM) (1–9/1,000,000 adult women) [1], ranges from 40% to 79% [2], but these figures do not fully capture the significant and debilitating reduction in life-quality experienced by most sufferers. While a number of organs can be affected, including the kidney and associated lymph nodes, it is the deterioration in lung function, which leads to disease progression. The early disease symptoms of LAM show high similarity to asthma, chronic obstructive pulmonary disease, and other obstructive lung diseases; therefore, the condition is frequently misdiagnosed [3]. Only through a combination of computer tomography, serological testing of increased serum vascular endothelial growth factor-D (VEGFD), and matrix metalloproteinase (MMP) levels, and lung biopsy, can clinicians confirm a LAM diagnosis with confidence [4]. Although the progression of the disease is relatively slow, most patients suffer from accelerating respiratory failure and can experience decades long dyspnea before lung transplantation is considered as a “last-resort” therapy.

LAM is caused by inherited (TSC-LAM) or acquired (sporadic or S-LAM) mutations of the tumor suppressor tuberous sclerosis complex (TSC) genes TSC1 (hamartin) or TSC2 (tuberin) [3]. The TSC1–TSC2 complex interacts with various signaling pathways and is also involved in regulation of the mechanistic target of Rapamycin (mTOR1) complex (mTORC1) via stimulation of GTPase activity of the small GTPase Rheb [5]. Although the majority of LAM tissues characteristically carry TSC1 or TSC2 mutations, a significant number of cases (10–15%) still present with no mutations in the TSC genes, suggesting undetected mutagenic events or deregulation of signaling pathways via an alternative route [6] which possibilities are actively researched in leading laboratories of the field [7].

Currently, treatment options remain limited to the mTOR inhibitor Rapamycin (Sirolimus) [8] that stabilizes lung function in most patients but does not offer progression-free survival.

As LAM occurs almost exclusively in women, some clinical studies have claimed that control of serum estrogen levels offers up an alternative route to preventing disease progression [9]. Although such attempts have mostly failed [9], we theorized that it was not the original idea but the study approach that might have been unsuccessful. In an attempt to understand more fully the broader disease mechanisms (in addition to TSC mutations) that might mediate LAM pathogenesis, we have performed TaqMan and Nanostring array analysis of LAM cells isolated directly from the lungs of transplant patients [10]. Through a combination of techniques, we have identified gene expression profiles and miRNA signatures that strongly indicate that an alteration in mitochondrial biogenesis and function are among the key determinants behind increased VEGF production and accelerated cell proliferation. To test our conclusions, we have also treated LAM cell lines with the potent, mito-targeting and mito-active drug candidate Proxison [11] and observed restoration of mitochondrial function and a corresponding reduction in VEGF production and proliferation capacity.

## Results

### Deregulation of nuclear receptors, vascularization, and miRNAs in LAM

**Fig. 1:**
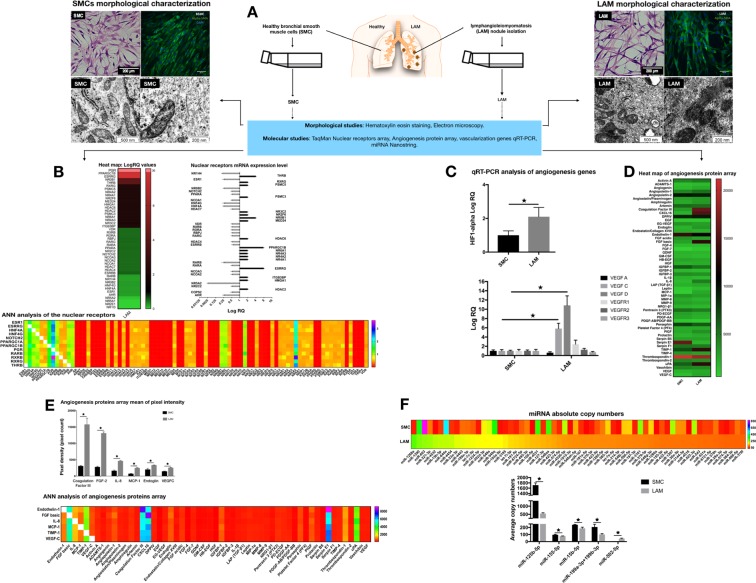
Morphological and molecular characterization of bronchial SMC controls and LAM cell lines. **a** Study rationale and cellular morphology. Normal bronchial SMC controls (*n* = 2) and patient-derived LAM cell lines (*n* = 4) were stained for hematoxylin eosin (magnification ×10, size-bar 200 μm) and for alpha-smooth muscle actin (ASMA) (ASMA green, DAPI blue, magnification ×40, size bar 40 μm). Electron microscopy of mitochondria in LAM cells and normal bronchial SMC controls (magnification 200 nm, the scale bar 500 nm). **b** Nuclear receptor TaqMan arrays n = 2 (data were generated from pooled samples of normal bronchial SMC controls *n* = 2, or patient derived LAM cell lines *n* = 4, respectively). Heat map of LogRQ values are shown. Nuclear receptor TaqMan data presented as LogRQ ± technical error of the replicates. ANN analysis of the nuclear receptor arrays was performed to demonstrate hidden interactions among different nuclear receptors. **c** Deregulation of VEGF expression in LAM samples. qRT-PCR analysis of genes affecting angiogenesis were performed and beta-actin was used as inner control. Data are presented as mean of log RQ ± SEM. Significant changes are marked as asterisk (*P* < 0.05); **d** Heat map of angiogenesis protein array. The figure presents mean of pixel intensity. **e** Angiogenesis array results of LAM cell lines *n* = 3 and normal SMC control *n* = 2 presented as mean of pixel intensity ± SEM. Significant changes are marked as asterisk (*P* < 0.05). ANN analysis of angiogenesis protein interaction hierarchy. **f** Analysis of 798 miRNA absolute copy numbers by Nanostring. miRNA copy numbers detected by Nanostring in pooled LAM (*n* = 4) and pooled, normal SMC (*n* = 2) samples. The heat map represents the most deregulated 141 miRNA in LAM samples compared to normal SMC controls. Copy number differences of specific miRNAs that are involved in mitochondrial biogenesis detected after Nsolver analysis were further analyzed in individual cell lines (normal SMCs (*n* = 2) and LAM (*n* = 4)). Data are presented as average copy number ± SEM, significant changes are marked as asterisk (*P* < 0.05)

Estrogen hormone affects cellular signaling and metabolism via two receptor types located on the cell membrane (Estrogen Receptor A or ERA) and within the nucleus (Estrogen Receptor B or ERB) [12]. A nuclear receptor array was performed using pooled samples of two individual bronchial smooth muscle cell (SMC) controls and pooled samples from four LAM patient-derived cell lines (Fig. 1a) (Supplementary Table 1 and Fig. 1). Of the 92 examined genes, 21 have shown an increase, and a further 30 a reduction in expression levels when compared to normal SMCs. The nuclear receptor TaqMan array (Fig. 1b) and consequent quantitative reverse transcription polymerase chain reaction (qRT-PCR) analysis on individual cell types (Supplementary Fig. 2) identified the progesterone receptor (PGR), the peroxisome proliferator-activated receptor gamma coactivator 1-beta (PPARGC1B) as being significantly overexpressed, and estrogen-related receptor gamma (ESRRG) as markedly upregulated. PPARGC1B is known to regulate the transcriptional activity of the estrogen receptor alpha (ERA), nuclear respiratory factor 1 (NRF1), and glucocorticoid receptor (GR) genes. The expression of each of these genes increased over seven-fold in the LAM cell lines tested (Fig. 1b). PPARGC1B overexpression is linked to increased mitochondrial number [13, 14], the active PRG isoform 4 to increased mitochondrial membrane potential and cellular respiration [13, 14], while ESRRG to control of mitochondrial biogenesis and energy metabolism [6, 14]. The significantly down-regulated nuclear receptor genes included NR5A2 and retinoic acid receptor beta (RARB). RARB is a member of thyroid-steroid hormone receptor superfamily (Fig. 1b) and both genes are known to play a powerful role in the inhibition of proliferation and stimulation of cellular differentiation [15]. Artificial Neural Network (ANN) analysis [16] of the data sets revealed a strong positive correlation with several retinoic acid receptors (RARB, RXRB, and RXRG) all of which bind the biologically active form of vitamin A and PGR (Fig. 1b). Changes in nuclear receptor gene expression in the disease cell lines directly point towards strong mitochondrial involvement in LAM linking data to previous studies demonstrating that LAM cell proliferation, driven by mTOR activation, requires major adjustments in energy metabolism [17]. Instead of utilizing NADP-driven oxidative phosphorylation, mitochondrial energy production by LAM sufferers (also seen in some cancers) is predominantly limited to aerobic glycolysis (Warburg effect) [17]. Such changes in energy metabolism lead to an increased expression of the hypoxia-inducible factor 1 alpha (HIF1-alpha) [17, 18] (Fig. 1c), and consequently to increase in VEGF expression [19]. In support, qRT-PCR analysis (Fig. 1c) and an angiogenesis protein array (Fig. 1c) detected strong up-regulation of LAM diagnostic markers VEGFC and VEGFD (Fig. 1c). A decrease in thrombospondin-1 (TSP1) and an increase in CXCL16 chemokine peptide levels were also observed in the LAM samples (Fig. 1d). These observations are particularly interesting as TSP1 is a recognized inhibitor of mitochondrial biogenesis [20], while CXCL16 regulates cellular invasion in non-small-cell lung cancer (NSCLC) [21]. ANN analysis of the angiogenesis array data (Fig. 1e) identified a strong association of CXCL16 and TSP1 with the fibroblast growth factor (FGF), Endothelin1, SerpinE1, and VEGFC (Fig. 1e) levels that are all involved in the stimulation of vascularization and the induction of myofibroblastic phenotype that in itself can reduce the capacity for pulmonary regeneration [22].

Further studies using a Nanostring methodology identified down-regulation of both the tumor suppressor miR125b-5p [23] and the low-density lipid oxidation induced autophagy regulator miR155-5p [24] in LAM. The apoptosis inducer miR-15b-5p [24] was down-regulated, while the cell proliferation and survival inducer miR-199a/b-3p [24] had increased copy numbers in individual LAM samples (Fig. 1f).

Based on the data above, compromised mitochondrial activity is appeared to be an additional factor in LAM disease progression.

### Mitochondrial dysfunction in LAM

**Fig. 2:**
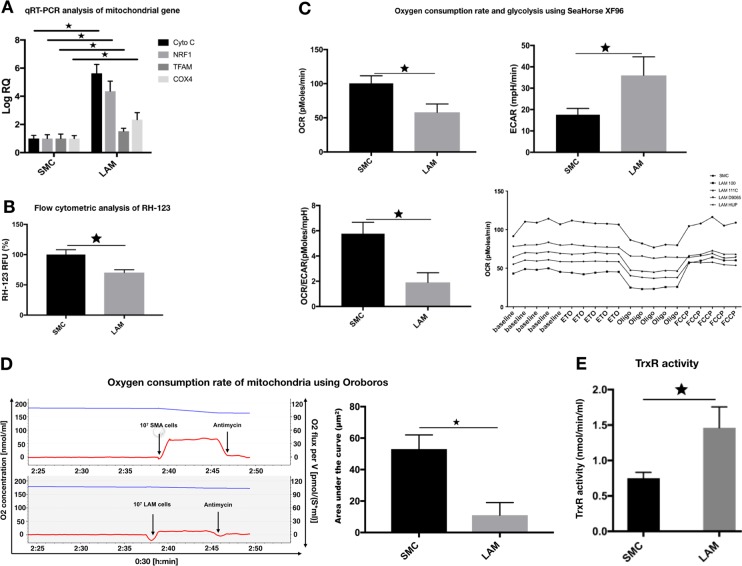
Altered function of mitochondria in LAM cells. **a** qRT-PCR analysis of mitochondrial gene expression in individual LAM cell lines (*n* = 4) compared to normal SMC controls (*n* = 2). Data are presented as mean log RQ ± SEM and significant changes are marked as asterisk (*P* < 0.05). **b** Flow cytometric analysis of RH-123 fluorescence intensity in individual LAM cell lines (*n* = 4) compared to normal SMC controls (*n* = 2). Data are presented as mean RFU ± SEM; significant changes are marked as asterisk (*P* < 0.05). **c** Oxygen consumption rate and glycolysis were measured by SeaHorse XF96 in individual LAM (*n* = 4) cell lines and normal SMC control cells (*n* = 2). Representative OCR and ECAR data are presented as mean ± SEM, significant changes are marked as asterisk (* *P* < 0.05). **d** Measurements of mitochondrial activity. Oxygen consumption rate of mitochondria measured using Oroboros (blue line = oxygen concentration, red line = oxygen flux per volume; quantification of oxygen consumption (area under the curve). **e** TrxR activity measured in individual LAM (*n* = 4) and normal, bronchial SMC control (*n* = 2) samples. Data are TrxR; activity is presented as mean of nmol min/ml ± SEM; significant changes are marked as asterisk (*P* < 0.05)

Electron microscopic images of the normal SMC and LAM cell lines showed a drastically different mitochondrial morphology (Fig. 1a) (Supplementary Fig. 3). Mitochondria in LAM cells were smaller, darker, and so electro-dense that the inner membrane cristae were not visible (Fig. 1a). The gene profiling data again support these microscopic observations. NRF1 encodes a homodimerizing protein, which functions as a transcription factor for key metabolic genes required for cellular growth, respiration, mitochondrial DNA transcription, and replication. NRF1 was higher in LAM than in control SMCs (Fig. 2a) and has previously [25] been linked to PPARGC1B gene expression (Fig. 1b). PPARGC1B in turn is responsible for constitutive non-adrenergic-mediated mitochondrial biogenesis via increased basal oxygen consumption [25], fat oxidation, non-oxidative glucose metabolism, and regulation of energy expenditure [25]. A pathway of biochemical events that seems to be confirmed here by the increase seen in HIF1 levels (Fig. 1c) and the corresponding overexpression of the VEGF gene family (Fig. 1c).

Additional markers of “mitochondrial health” showed significant changes. The mitochondrial transcription factor A (TFAM) that encodes a protein critical in both mitochondrial DNA repair and replication was higher in diseased LAM cell lines than in normal SMC controls (Fig. 2a). The observed alterations in transcription levels appeared to impact on all aspects of mitochondrial function. Both CytoC (cytochrome complex), an inner membrane protein of the mitochondria that is an essential component of the electron transport chain [26], as well as Cox4 (cytochrome *c* oxidase) that catalyzes oxygen reduction [26] were significantly elevated (Fig. 2a).

To investigate overall mitochondrial activity, a combination of flow cytometric analysis of the mitochondrial membrane Rhodamine-123 (RH-123) a cell-permeable, cationic, green-fluorescent dye, Oroboros, and Seahorse analyses were performed. Mitochondrial energization induces quenching of RH-123 fluorescence and the rate of fluorescence decay is proportional to mitochondrial membrane potential. Based on this analysis, LAM mitochondrial membrane activity is twice as high as that seen in normal, SMC controls (Fig. 2b). In contrast, increased glycolysis and reduced oxidation was detected by metabolic analysis of LAM cells using Seahorse (Fig. 2c) and Oroboros technology (Fig. 2d). At first sight, these biochemical results may appear contradictory, however, during reductive stress, when electron acceptors are expected to be mostly reduced, some redox proteins can donate electrons to O_2_ instead. This process can increase net mitochondrial reactive oxygen species (ROS) production, despite the concomitant enhancement of ROS scavenging systems [27]. For example, normally antioxidant matrix NADPH reductases, together with glutathione reductases and thioredoxin reductases (TrxR) [28], can all go on to generate H_2_O_2_ by leaking electrons from their reduced flavoprotein to O_2_. Generation of this net mitochondrial ROS spill-over can cause oxidative injury and can critically damage mitochondria. The process by which cells remove these damaged or dysfunctional mitochondria is known as mitophagy. Any damage to the mitophagy process may result in abnormal mitochondrial function. To test this theory, TrxR activity was determined in both normal, SMCs, and LAM cell lines. In the latter, TrxR activity was significantly higher than in normal SMC controls (Fig. 2e).

Interestingly, the Trx2–TrxR2 system has been reported to be an anti-angiogenic target of auranofin, a redox enzyme inhibitor gold complex [28]. The high affinity of auranofin for thiol and selenol groups and through the inhibition of redox enzymes such as TrxR can modify the redox balance in mitochondria [28]. Our studies reported here, together with the above supporting evidence from the published literature, we theorized that drugs that can inhibit TrxR activity and restore normal mitochondrial function might be able to reduce LAM progression.

### Mitochondria as a potential therapeutic target in LAM

**Fig. 3:**
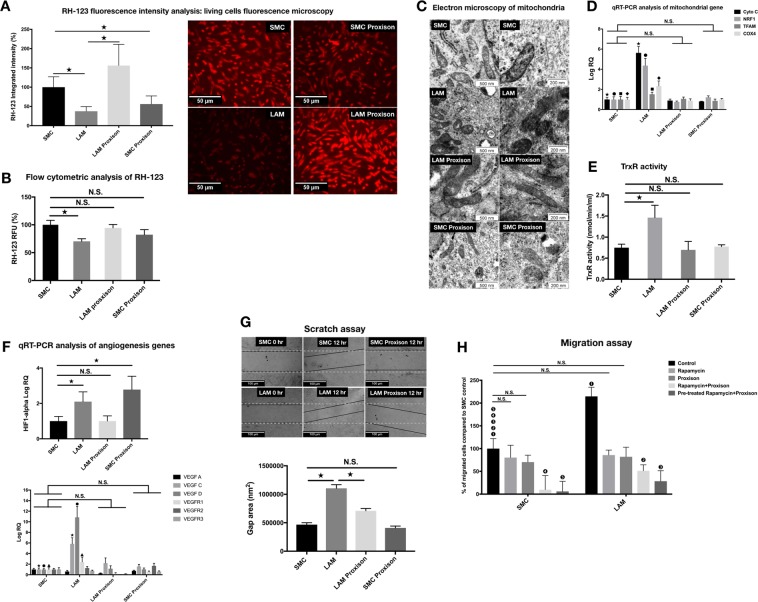
Proxison normalizes mitochondrial morphology and function in LAM cells. **a** Proxison (3 µM, 1 h)-treated normal SMC and LAM cells were incubated with 2.5 µM RH-123 and then fluorescence microscopy was used to analyze fluorescence intensity (magnification ×20, scale bar 50 µm). Quantification of fluorescence intensity in living cells was performed using ImageJ software. Data are presented as pixel intensity ± SEM; significant changes are marked as asterisk (*P* < 0.05). **b** Proxison (3 µM, 1 h)-treated normal SMC and LAM cells were incubated with 2.5 µM RH-123 and then fluorescence was analyzed by flow cytometry. Data are presented as mean of RFU ± SEM; significant changes marked as * *P* < 0.05. **c** Representative morphological changes in the mitochondria of LAM cell lines following Proxison treatment. Electron microscopy of mitochondria of untreated and Proxison (3 µM, 1 h)-treated LAM cells and normal SMC control cells (scale bars are 500 and 200 nm, respectively). **d** qRT-PCR analysis of mitochondrial gene expression in untreated and Proxison (3 µM, 1 h) treated LAM cell lines (*n* = 4) compared to normal SMC controls (*n* = 2); Data are presented as mean log RQ ± SEM and significant changes are marked as asterisk, solid circle, solid rhombus, and solid square (*P* < 0.05). **e** TrxR activity of Proxison (3 µM, 1h)-treated LAM cell lines (*n* = 4) compared to normal SMC controls (*n* = 2). TrxR activity is presented as mean ± SEM and significant changes are marked as asterisk (*P* < 0.05). **f** qRT-PCR analysis of angiogenesis related gene expression in untreated and Proxison (3 µM, 1 h)-treated cell cultures (LAM *n* = 4, normal bronchial SMC *n* = 2). Data are presented as mean RQ ± SEM and significant changes are marked as asterisk, solid circle, and solid triangle (in all results significance was *P* < 0.05). **g** Proliferation capacity following Proxison treatment (*n* = 3 technical repeats). Representative pictures of scratch assays in untreated and Proxison (3 µM, 1 h)-treated LAM cell lines (*n* = 4) compared to normal SMC controls (*n* = 2) after 12 h incubation. Data are presented as mean of cell growth (gap) area nm^2^ ± SEM; significant changes are marked as asterisk (*P* < 0.05). **h** Migration capacity of LAM cell lines. LAM cell lines (*n* = 2) and normal SMCs (*n* = 2) were treated with Rapamycin (20 nM, 24 h), Proxison (3 µM, 24 h), Rapamycin (20 nM, 24 h)+Proxison(3 µM, 24 h), and finally cells were pre-treated with Rapamycin for 48 h (20 nM/24 h) and then incubated with Proxison (3 µM, 24 h). Images are presented as the number of cells migrated through the membrane to the lower side of the chamber and were stained with DAPI. Data are presented as the percentage of migrated LAM cells compared to normal SMC ± SEM and significant changes are marked as (**1**), (**2**), (**3**), (**4**) and (**5**) (in all results significance was *P* < 0.05)

The novel synthetic flavonoid, Proxison (7-decyl-3-hydroxy-2-(3,4,5-trihydroxyphenyl)-4-chromenone) (Antoxis Ltd, UK), is a potent antioxidant accessing the mitochondria [11]. Proxison combines key structural attributes of the natural flavonoid myricetin [29], with a strategically placed lipophilic chain to effectively protect cell membranes from lipid peroxidation [11, 29]. To test the effects of Proxison, both normal SMCs and LAM cell lines were treated with the drug (Supplementary Fig. 4). Mitochondrial activity was measured using RH-123 (Fig. 3a, b) and TrxR activity (Fig. 3e). Both tests showed striking normalization of mitochondrial function in LAM cell lines while Proxison appeared to have had little or no effect on normal SMCs. Rapid improvement was detected in the mitochondrial morphology of LAM cells with cristae of the inner membrane becoming visible again by electron microscopy (Fig. 3c). Morphological changes were associated with the reduced gene expression of CytoC, NRF1, TFAM, and Cox4 (Fig. 3d) as well as with gene expression of VEGF ligands and receptors falling back to normal levels (Fig. 3f). Additional functional studies, scratch and migration assays have shown that Proxison treatment reduced the proliferation (Fig. 3g) and migration capacity (Fig. 3h) of LAM cells and such effect was additive to Rapamycin treatment in both gene expression and cellular migration (Supplementary Figs 5 and 6).

## Discussion

While earlier publications may have hinted at the importance of energy metabolism in the pathogenesis of LAM [6], this is the first detailed molecular analysis of patient-derived LAM cell lines, allowing the assessment of mitochondrial function and biogenesis to be defined in the pathomechanism of LAM, and its utility as a novel target for therapy investigated.

Mitochondrial biogenesis is under multifactorial regulation with hormones such as estrogen [30] known to have a profound effect on activity. This complex biochemistry is coordinated through a network of transcriptional coactivators such as PPARGC1A and PPARGC1B [31] together with PPARs (peroxisome proliferator-activating receptors), ERRs (estrogen-related receptors), and NRF1 hormones and in concert they are able to influence and control aspects of energy metabolism [32]. Gene profiling has confirmed that the expression of all these nuclear receptors and/or coactivators are upregulated in LAM pathology. Taken together these data strongly support the conclusion that mitochondrial malfunction has a key role in LAM disease. Experiments with the mitochondria targeting Proxison, a novel pre-clinical drug candidate [11, 29, 33] appeared to sufficiently “heal” the critically damaged mitochondria in LAM. In migration assays the inhibitory effects of Proxison were additive to Rapamycin which result needs further evaluation.

**Fig. 4:**
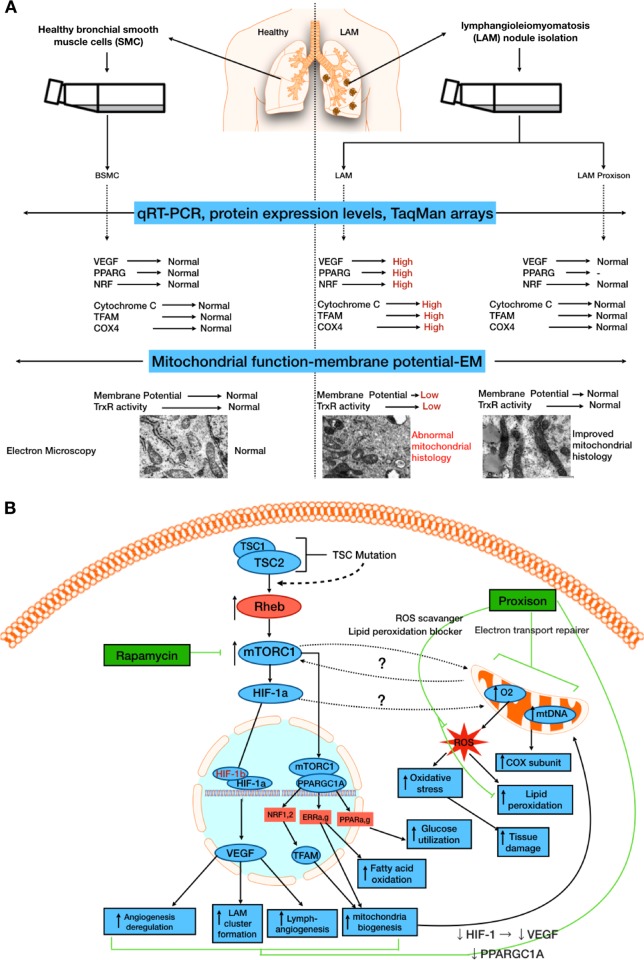
**a** Summary of LAM pathomechanism. **b** Summary of signaling pathway interactions in LAM revealing current and future therapeutic targets. The study led to the identification of mitochondrial dysfunction in LAM. Treatment with the mito-active candidate drug Proxison encouraged reestablishing the homeostasis in a diverse range of key pathways including VEGF and TFAM. Rapamycin, by acting directly on mTORC1, may also indirectly affect mitochondrial metabolism (as well as VEGF and TFAM), while Proxison, acting directly on the mitochondria, may indirectly influence the mTORC1 pathway. In migration assays the effects of the two drugs, Rapamycin and Proxison, were additive, indicating that from a clinical perspective there is a possibility of a combination therapy aimed at two different, but interacting facets of the disease process providing the best outcome for patients

In summary, we believe our study added a novel angle to the current understanding of the condition LAM and have proposed through Fig. 4 how the original importance of mTORC1 [6], and its links to disease progression, fits alongside the new hypothesis that mitochondrial metabolism is an additional therapeutic target.

## Materials and methods

Lung tissue samples were obtained from human lung transplant donors, in accordance with the Declaration of Helsinki and approved by the Institutional Review Board at the University of Pennsylvania [5]. Four patient derived cell lines LAM-100, LAM-111C, LAM-D9065, and LAM-HUP were used in the present study. Controls were primary, normal human bronchial SMCs (Lonza, Basel, Switzerland). Electron microscopy on 90-nm-thick sections were performed using a Jeol 1200 and Jeol 1400 transmission electron microscope (Jeol Ltd, Tokyo, Japan) at 80 kV. Images were acquired using an integrated MegaView III digital camera (Olympus Soft Imaging Solutions GmbH, Munster, Germany). Flow cytometry was performed on Rhodamine-123 (RH-123) (Sigma-Aldrich, St Louis, MO, USA)-treated cell suspensions using a FACS Canto II flow cytometer (BD Immunocytometry Systems, Erembodegen, Belgium). Fluorescence microscopy images were acquired by an Olympus IX-81 (OLYMPUS Corporation, Tokyo, Japan) light and fluorescent microscope. RNA was isolated with MN NucleoSpin RNA isolation kit (Macherey-Nagel, Düren, Germany). RNA concentration was measured using NanoDrop (Thermo Fisher Scientific, Waltham, USA). Human Nuclear Receptors TaqMan®Array (Thermo Fisher Scientific, Waltham, USA). TaqMan PCR reaction was performed using ABI StepOnePlus system and data were analyzed with StepOne software. MicroRNA expression was normalized to U6 expression. Nanostring assay was analyzed using the nCounter Analysis System (NanoString Technologies, Washington, USA). Angiogenesis was assessed using a Human Angiogenesis Array Kit (R&D Systems, Minneapolis, USA). Protein concentration was determined using a fluorescent protein assay (Qubit protein; Thermo Fisher Scientific, Waltham, USA). Quantitative RT-PCR was performed using SensiFAST SYBR Green reagent (BioLine, London, UK) in an ABI StepOnePlus system (Thermo Fisher Scientific, Waltham, USA) and data were analyzed with StepOne software and normalized to beta-actin as a housekeeping gene and calculated according to the 2^−ddCt^ method. Array data were evaluated using a feedforward artificial neural network (ANN) (Neurosolutions 6; NeuroDimension Inc.) software. Metabolic profiling was performed using *SeaHorse XF96* (Agilent Technologies, USA) [11] and *Oroboros* (O2k, OROBOROS Instruments, Innsbruck, Austria) platforms [12]. Transwells were used for migration assay (Costar, Corning Incorporated, Sigma-Aldrich, St Louis, MO, USA). TRXR activity was assessed using a Thioredoxin Reductase Assay Kit (Abcam, Cambridge, MA, USA). Statistical analysis was performed using the independent samples *t*-test and one-way ANOVA with Bonferroni correction. *P* < 0.05 was considered as significant. For extensively detailed Materials and methods refer to Full Methods (Supplementary Material).

## Supplementary Information

Supplemental Material

Individual data points

## References

[1] Harknett EC, Chang WYC, Byrnes S, Johnson J, Lazor R, Cohen MM (2011). Use of variability in national and regional data to estimate the prevalence of lymphangioleiomyomatosis. QJM.

[2] Hayashida M, Seyama K, Inoue Y, Fujimoto K, Kubo K (2007). The epidemiology of lymphangioleiomyomatosis in Japan: a nationwide cross-sectional study of presenting features and prognostic factors. Respirology.

[3] Astrinidis A, Khare L, Carsillo T, Smolarek T, Au KS, Northrup H (2000). Mutational analysis of the tuberous sclerosis gene TSC2 in patients with pulmonary lymphangioleiomyomatosis. J Med Genet.

[4] Chang William YC, Cane Jennifer L, Blakey John D, Kumaran Maruti, Pointon Kate S, Johnson Simon R (2012). Clinical utility of diagnostic guidelines and putative biomarkers in lymphangioleiomyomatosis. Respiratory Research.

[5] Goncharova EA, Goncharov DA, Eszterhas A, Hunter DS, Glassberg MK, Yeung RS (2002). Tuberin regulates p70 S6 kinase activation and ribosomal protein S6 phosphorylation: a role for the TSC2 tumor suppressor gene in pulmonary lymphangioleiomyomatosis (LAM). J Biol Chem.

[6] Krymskaya VP, McCormack FX (2017). Lymphangioleiomyomatosis: a monogenic model of malignancy. Annu Rev Med.

[7] Julian LisaM, Delaney SeanP, Wang Ying, Goldberg AlexanderA, Doré Carole, Yockell-Lelièvre Julien (2017). Human pluripotent stem cell-derived TSC2-haploinsufficient smooth muscle cells recapitulate features of lymphangioleiomyomatosis. Cancer Res.

[8] MacKeigan JP, Krueger DA (2015). Differentiating the mTOR inhibitors everolimus and sirolimus in the treatment of tuberous sclerosis complex. Neuro Oncol.

[9] Lu C, Lee HS, Pappas GP, Dilling DF, Burger CD, Shifren A (2017). A phase II clinical trial of an aromatase inhibitor for postmenopausal women with lymphangioleiomyomatosis. Ann Am Thorac Soc.

[10] Goncharova EA, Goncharov DA, Lim PN, Noonan D, Krymskaya VP (2006). Modulation of cell migration and invasiveness by tumor suppressor TSC2 in lymphangioleiomyomatosis. Am J Respir Cell Mol Biol.

[11] Drummond NJ, Davies NO, Lovett JE, Miller MR, Cook G, Becker T (2017). A synthetic cell permeable antioxidant protects neurons against acute oxidative stress. Sci Rep.

[12] Barros RPA (2011). Estrogen receptors and the metabolic network. Cell Metab.

[13] Shao D, Liu Y, Liu X, Zhu L, Cui Y, Cui A (2010). PGC-1β-regulated mitochondrial biogenesis and function in myotubes is mediated by NRF-1 and ERRα. Mitochondrion.

[14] Hayashi K, Yokozaki H, Naka K, Yasui W, Lotan R, Tahara E (2001). Overexpression of retinoic acid receptor beta induces growth arrest and apoptosis in oral cancer cell lines. Jpn J Cancer Res.

[15] Gyftopoulos K, Sotiropoulou G, Varakis I, Barbalias GA (2000). Cellular distribution of retinoic acid receptor-alpha; in benign hyperplastic and malignant human prostates: comparison with androgen, estrogen and progesterone receptor cStatus. Eur Urol.

[16] Motalleb G (2014). Artificial neural network analysis in preclinical breast cancer. Cell J.

[17] Liberti MV, Locasale JW (2016). The Warburg effect: how does it benefit cancer cells?. Trends Biochem Sci.

[18] Marín-Hernández A, Gallardo-Pérez JC, Ralph SJ, Rodríguez-Enríquez S, Moreno-Sánchez R (2009). HIF-1alpha modulates energy metabolism in cancer cells by inducing over-expression of specific glycolytic isoforms. Mini Rev Med Chem.

[19] Simiantonaki N, Jayasinghe C, Michel-Schmidt R, Peters K, Hermanns MI, Kirkpatrick CJ (2008). Hypoxia-induced epithelial VEGF-C/VEGFR-3 upregulation in carcinoma cell lines. Int J Oncol.

[20] Lawler PR, Lawler J (2012). Molecular basis for the regulation of angiogenesis by thrombospondin-1 and -2. Cold Spring Harb Perspect Med.

[21] Hald SM, Kiselev Y, Al-Saad S, Richardsen E, Johannessen C, Eilertsen M (2015). Prognostic impact of CXCL16 and CXCR6 in non-small cell lung cancer: combined high CXCL16 expression in tumor stroma and cancer cells yields improved survival. BMC Cancer.

[22] Kovacs T, Csongei V, Feller D, Ernszt D, Smuk G, Sarosi V (2014). Alteration in the Wnt microenvironment directly regulates molecular events leading to pulmonary senescence. Aging Cell.

[23] Mei LL, Wang WJ, Qiu YT, Xie XF, Bai J, Shi ZZ (2017). miR-125b-5p functions as a tumor suppressor gene partially by regulating HMGA2 in esophageal squamous cell carcinoma. PLoS ONE.

[24] Gozuacik D, Akkoc Y, Ozturk DG, Kocak M (2017). Autophagy-regulating microRNAs and cancer. Front Oncol.

[25] Miglio G, Rosa AC, Rattazzi L, Collino M, Lombardi G, Fantozzi R (2009). PPARγ stimulation promotes mitochondrial biogenesis and prevents glucose deprivation-induced neuronal cell loss. Neurochem Int.

[26] Hüttemann M, Pecina P, Rainbolt M, Sanderson TH, Kagan VE, Samavati L (2011). The multiple functions of cytochrome c and their regulation in life and death decisions of the mammalian cell: from respiration to apoptosis. Mitochondrion.

[27] Fernández-Vizarra E, Tiranti V, Zeviani M (2009). Assembly of the oxidative phosphorylation system in humans: what we have learned by studying its defects. Biochim Biophys Acta.

[28] Rigobello MP, Scutari G, Boscolo R, Bindoli A (2002). Induction of mitochondrial permeability transition by auranofin, a Gold(I)-phosphine derivative. Br J Pharmacol.

[29] Bennett CJ, Caldwell ST, McPhail DB, Morrice PC, Duthie GG, Hartley RC (2004). Potential therapeutic antioxidants that combine the radical scavenging ability of myricetin and the lipophilic chain of vitamin E to effectively inhibit microsomal lipid peroxidation. Bioorg Med Chem.

[30] Lin J, Handschin C, Spiegelman BM (2005). Metabolic control through the PGC-1 family of transcription coactivators. Cell Metab.

[31] Uldry M, Yang W, St-Pierre J, Lin J, Seale P, Spiegelman BM (2006). Complementary action of the PGC-1 coactivators in mitochondrial biogenesis and brown fat differentiation. Cell Metab.

[32] Finck BN, Kelly DP (2007). Circulation.

[33] Moini H, Arroyo A, Vaya J, Packer L (1999). Bioflavonoid effects on the mitochondrial respiratory electron transport chain and cytochrome *c* redox state. Redox Rep.

