# Supplementary material for: Mitochondrial dysfunction is a key determinant of the rare disease lymphangioleiomyomatosis and provides a novel therapeutic target
Source: Oncogene. 2018 Dec 20;38(16):3093–101. doi: 10.1038/s41388-018-0625-1 (PMC6484686; doi:10.1038/s41388-018-0625-1)
Supplement: Supplementary file 1 — Supplemental Material [file 41388_2018_625_MOESM1_ESM.pdf]

SUPPLEMENTAL MATERIAL

MATERIALS AND METHODS

Ethical Statement

Lung tissue samples were obtained from human lung transplant donors, in accordance with the Declaration of Helsinki and approved by the Institutional Review Board at the University of Pennsylvania (1). Human tissue was obtained from National Disease Research Interchanged (NDRI, Philadelphia, PA, USA). LAM patients had given written consent and all the collected samples were treated anonymously.

LAM cell lines, primary, bronchial Smooth Muscle Cells (SMC) and cell culture conditions

Primary cultures of human LAM cells where established in the Department of Medicine, University of Pennsylvania, Pennsylvania, USA (1). Briefly, the primary cultures of LAM cells were dissociated from the LAM nodules of transplant patients. Each LAM nodule was used to establish individual cell lines (characterized based on alpha smooth muscle actin (ASMA) expression, mTORC1 activation, HMB45 immunoreactivity, DNA synthesis, TSC2-mTOR-PS6-MLANA genes, and cell migration) (1, 2). In the current study LAM cell lines were derived from individual patients and identified as LAM-100, LAM-111C, LAM-D9065 and LAM-HUP. As controls, primary cultures of normal, human bronchial smooth muscle cells (SMC), were purchased from Lonza (Basel, Switzerland). Normal, bronchial SMC and LAM cells were cultured at 37°C, 5% CO_2 in SMC Growth Medium (Lonza, Basel, Switzerland). Primary, normal, bronchial SMC cells from two individual donors and the four individual cell lines were characterized using ASMA staining and testing various (PS6, MLANA, mTOR) gene expressions (Supplemental figure 1, Supplemental table1).

Hematoxylin eosin staining

Cytospins of normal, bronchial SMC and LAM cell lines were stained in Mayer's hematoxylin solution (Sigma-Aldrich, St. Louis, USA) for 10 min, washed, then differentiated with 0.25% acetic acid and in eosin solution. Sections were mounted using Vectashield mounting medium (Vector Laboratories, Burlingame, USA). Images were taken using Nikon Eclipse Ti-U inverted microscope.

Electron microscopy

Cells were resuspended in 2.5% glutaraldehyde in 0.1 M sodium-cacodylate buffer (pH 7.4) for 24h, rinsed in 0.1 M sodium-cacodylate buffer and pelleted. The pellet was embedded in Spurr low-viscosity resin with ERL 4221 used as the epoxy monomer and cured at 70°C for 16h. For transmission electron microscopy (TEM), 90 nm thick sections were stained with alcoholic uranyl-acetate and Reynolds lead-citrate and examined using Jeol 1200 and Jeol 1400 transmission electron microscope (Jeol Ltd, Tokyo, Japan) at 80 kV. Images were acquired using an integrated MegaView III digital camera (Olympus Soft Imaging Solutions GmbH; Munster, Germany).

Proxison treatment

Normal, bronchial SMC and LAM cell cultures were treated with 3 µM Proxison (Antoxis Ltd, Aberdeen, UK) for 1h at 37°C, 5% CO_2. In Proxison and Rapamycin combination treatment (migration assay, mitochondrial genes qRT-PCR), cells were treated with 3 µM Proxison and 20 nM Rapamycin for 24 h at 37°C, 5% CO_2.

Rapamycin treatment

Normal, bronchial SMCs and LAM cell cultures were treated with 20 nM Rapamycin catalog: tlrl-rap (InvivoGen, San Diego, USA)for 24h at 37°C, 5% CO_2 in monotherapy. Rapamycin pre-treatment was made for 48h (20nM/24h) then 3 µM Proxison was added for an extra 24h.

Flow cytometry

Normal, bronchial SMC and LAM cells (100,000) were collected from Proxison treated and control cultures. Cell cultures were incubated with 2.5 µM of Rhodamine 123 (RH-123) (Sigma) for 30 min at 37°C, then cooled to 4°C and washed twice with PBS. Viability was tested using propidium iodide (PI) staining (Invitrogen, Ltd). Cells were analyzed using FACS Canto II flow cytometer (BD Immunocytometry Systems, Erembodegen, Belgium) with BD FACS DIVA software V6 and data were analyzed by FCS Express V3 software.

RH-123 fluorescence microscopy

Normal SMC and LAM cells were cultured for 3 days using Falcon™ chambered cell culture slides (Thermo Fisher Scientific, Waltham, USA), then treated with Proxison as described above. Images from living cells were acquired using an Olympus IX-81 (OLYMPUS Corp., Tokyo, Japan) light and fluorescent microscope, then densitometry was performed.

Immunofluorescent staining

Normal, bronchial SMC and LAM cells were cultured for 3 days using Falcon™ chambered cell culture slides (Thermo Fisher Scientific, Waltham, USA). Cell cultures were then fixed with 4% formaldehyde and permeabilized with PBS containing 0.1% Triton-X and 5% BSA. Anti-alpha - Smooth Muscle Actin Antibody catalog: MAB1420 (R&D Systems) (1∶100) and anti-mouse Alexa 488 catalog: A28175 (Thermo Fisher Scientific, Waltham, USA) (1:200) were used for immunofluorescent staining. Nuclei were counter stained with DAPI. Images were acquired using an Olympus IX-81 (OLYMPUS Corporation, Tokyo, Japan) both light and fluorescence microscope.

Migration assay

200,000 cells of both normal SMC and LAM cell lines were seeded in serum-free media using various treatments in the upper chamber of the Transwell migration plate. The pore size of the membrane was 8.00µm (24-well format, Costar, Corning Incorporated). The chambers were incubated at 37°C for 16 h. Chambers were fixed in PBS containing 4% paraformaldehyde, stained with DAPI and membranes were mounted to microscopic slides (supplemental figure 6). Images were acquired using an Olympus IX-81 (OLYMPUS Corporation, Tokyo, Japan). The number of migrated cells was analyzed using ImageJ particular analyzer.

RNA isolation

Total RNA was extracted from normal SMC and LAM cell cultures with MN NucleoSpin RNA isolation kit according to the manufacturer's protocol (Macherey-Nagel, Düren, Germany). The concentration of RNA samples was measured using NanoDrop (Thermo Fisher Scientific, Waltham, USA).

Total RNA from human lung tissues were obtained using TRIzol reagent (Invitrogen, Thermo Fisher Scientific, Waltham, USA). RNA (1 µg) was digested with DNase (Sigma-Aldrich, St. Louis, USA) to avoid DNA contamination.

TaqManArray, Nanostring and Quantstudio chips

Human Nuclear Receptors TaqMan®Array

cDNA was synthesized with high capacity RNA to cDNA kit (Thermo Fisher Scientific, Waltham, USA). Reverse transcription was performed with random hexamer primers. Each sample was mixed with TaqMan Universal Master Mix (Thermo Fisher Scientific, Waltham, USA). TaqMan PCR

reaction was performed using ABI StepOnePlus system and data were analyzed with StepOne software. MicroRNA expression was normalized to U6 expression.

Nanostring

100 ng of total RNA/cell culture was isolated and analyzed using the nCounter Analysis System (NanoString Technologies) and the nCounter Human v2 miRNA Panel containing 798 unique miRNA barcodes. Copy count assay was performed using the Nanostring ncounter SPRINT. Analysis was performed using the Nsolver software.

Quantstudio 12k flex

cDNA was prepared using TaqMan miRNA reverse transcriptase kit and Megaplex RT primers Pool A and B (Thermo Fisher Scientific, Waltham, USA) according to manufacturers' protocol using 350ng-1000ng of total RNA as starting material. miRNA expression levels were performed using open array miRNA card Pool A and B and Quantstudio 12k flex (Thermo Fisher Scientific, Waltham, USA).

Protein array

Angiogenesis array

Cell lysates of 1 x 10^7 cells/ml were assessed using a Human Angiogenesis Array Kit (R&D Systems, Minneapolis, USA). Protein concentration was determined using a fluorescent protein assay (Qubit Protein, Thermo Fisher Scientific, Waltham, USA). Briefly, the Detection Antibody Cocktail was mixed with each sample and incubated with the membrane at 4°C overnight, then with Streptavidin-HRP at room temperature finally with the Chemiluminescent-Reagent Mix. Images were captured using LAS-4000 (GE Healthcare Bio-Sciences AB Uppsala, Sweden), and intensity was determined using ImageJ (https://imagej.nih.gov/ij/) and normalized to the reference spots.

Quantitative qRT-PCR

cDNA was synthesized as described above. qRT-PCR was performed using SensiFAST SYBR Green reagent (BioLine, London, UK) in an ABI StepOnePlus system. Gene expressions using sequence specific primers (Table 1) were analyzed with StepOne software and normalized to beta-actin as a housekeeping gene. Changes in gene expression were calculated according to the 2^{-ddCt} method.

Gene name (abbreviation) Accession code	Forward primer	Reverse primer
beta-actin NM_001101.4	GCGCGGCTACAGCTTCA	CTTAATGTCACGCACGATTTCC
NRF1 NM_005011.4	CAGCCGCTCTGAGAACTTCA	TTCCCGCCCATGCTGTTTAT
Cyt C NM_018947.5	TCAGGCCCCTGGATACTCTT	AAGTCTGCCCTTTCTTCCTTC
COX4 NM_001318797.1	GTTTCACCGCGCTCGTTATC	TTGGCCACCCACTCTTTGTC
VEGFD NM_004469.4	GAACACCAGCACCTCGTACA	ACAGACACACTCGCAACGAT
VEGFC NM_005429.4	CCCGCCTCTCCAAAAAGCTA	TGGACACAGACCGTAACTGC
VEGFA NM_001204384.1	TTCTGGGCTGTTCTCGCTTC	TTGTCACATACGCTCCAGGAC
VEGFR1 NM_017020485.1	ACCATACCTCCTGCGAAACC	TCAGAGGCCCTTTCAGCATT
VEGFR2 NM_002253.3	CGGTCAACAAAGTCGGGAGA	CAGTGCACCACAAAGACACG
VEGFR3 NM_001354989.1	TGTACACCACGCAGAGTGAC	AGCCTTTGTAGGTCGTTGGG
TFAM NM_003201.2	CTCCGCAGGCTAGAGGATTG	CAGCTTTTCCTGCGGTGAAT

TSC1<br>NM_001162427.1	TGCTGTACGTCCAAGATGGC	TTACAAGCATAGGGCCACGG
TSC2<br>NM_001363528.1	GGCGGCACAGAACTACAACT	GGAATTCCGAGGACAAGCCA
HIF1-alpha<br>NM_001530.3	GTCTGAGGGGACAGGAGGAT	GCACCAAGCAGGTCATAGGT
ESR1<br>NM_000125.3	GACTGCACTTGCTCCCGT	CCACTTCGTAGCATTTGCGG
THRB<br>NM_001354712.1	AGGGCACTGGTAATTTGGCT	TGGCTTTGTCACCACACACT
NR0B1<br>NM_000475.4	GACTGTGGAAGTCTCGGAGC	ACTTGATGGCTTGGACCTGG
NR5A2<br>NM_205860.2	CCCAAGGCCACGAAATTTGA	GCCCAGCACCAATAGGTGTAA
ESRRG<br>NM_001438.3	AATAATGGTTGCCGGTCGCA	TGCAGAGAAGCTCTTCCTCGTAG
AHR<br>NM_001621.4	CCACTTCAGCCACCATCCAT	AAGCAGGCGTGCATTAGACT
MLANA<br>NM_005511.1	CTGCTCATCGGCTGTTGGTA	GAGACACTTTGCTGTCCCGA
MTOR<br>NM_004958.3	TTGAGGTTGCTATGACCAGAGAGAA	TTACCAGAAAGGACACCAGCCAATG
RPS6<br>NM_001010.2	TGTTACTCCACGTGTCCTGC	AAGTCTGCGTCTCTTCGCAA
RARB<br>NM_000965.4	ATGATTCGGGGCTGGGAAAAA	AGGGTAAGGCCGTCTGAGAA
PGR<br>NM_001202474.3	TGCCTGAAGTTTCGGCCATA	AAGCGGGAATCTTCCTTGGG

Supplemental Table 1.: Quantitative qRT- PCR primers.

Artificial neural network (ANN) analysis

Gene expression data of nuclear receptors and angiogenesis protein array was evaluated using a feed forward artificial neural network (ANN) (Neurosolutions 6, NeuroDimension Inc.) software (5-9).

Metabolic profiling

Metabolic profiling using SeaHorse XF96

Normal SMC and LAM metabolic profiles were generated using the Seahorse X96 platform (Agilent Technologies, USA) (3). Briefly, cells were plated into Seahorse cell plates, then at 90% confluence and after recording baseline oxygen consumption, cells were treated with butyril-cAMP (500 µM), oligomycin (2 µM) and antimycin (10 µM). Antimycin-resistant oxygen consumption was considered as. Baseline oxygen consumption and membrane leak (OCR after oligomycin treatment) was calculated. Glycolysis was assessed through the extracellular acidification value (ECAR) and ECAR/OCR values were calculated.

Metabolic profiling using Oroboros

LAM and normal SMCs respiration was measured using a high resolution Oxygraph-2k (O2k, OROBOROS Instruments, Innsbruck, Austria) (4). Oxygraph-2k chambers were filled with respiration medium and the chamber was allowed to equilibrate and the baseline built. Cells (10,000,000) in smooth muscle growth medium from both SMC and LAM were injected into the chamber. To generate a closed system Antimycin was inserted to stop cellular oxygen consumption (4).

TRXR activity

Cell lysates (1 x 107 cells/Ml) were collected to assess TrxR activity using a Thioredoxin Reductase Assay Kit (Abcam, Cambridge, MA, USA). Protein concentration was determined (Qubit Protein, Thermo Fisher Scientific, Waltham, USA), then a TrxR activity assay was performed according to the manufacturer's instructions. OD was measured at 412 nm.

Statistical analysis

Statistical analysis was performed with SPSS version 20 software. Data were generated from an average of 3 technical repeats on LAM (n=4) compared to normal SMC (n=2) ± standard error of mean (SEM) are presented, and statistical analysis was performed using the independent samples t-test and one-way ANOVA with Bonferroni correction. $p<0.05$ was considered as significant. For detailed variation within each sample refer to supplementary pdf individual data point file.

References

1. Goncharova EA, Goncharov DA, Eszterhas A, Hunter DS, Glassberg MK, Yeung RS *et al.* Tuberin regulates p70 S6 kinase activation and ribosomal protein S6 phosphorylation: A role for the TSC2 tumor suppressor gene in pulmonary lymphangioleiomyomatosis (LAM). *J Biol Chem* 2002; **277**: 30958–30967.
2. Goncharova EA, Goncharov DA, Lim PN, Noonan D, Krymskaya VP. Modulation of cell migration and invasiveness by tumor suppressor TSC2 in lymphangioleiomyomatosis. *Am J Respir Cell Mol Biol* 2006; **34**: 473–480.
3. Kristóf E, Doan-Xuan Q-M, Bai P, Bacso Z, Fésüs L. Laser-scanning cytometry can quantify human adipocyte browning and proves effectiveness of irisin. *Sci Rep* 2015; **5**: 12540.
4. Pesta D, Gnaiger E. High-Resolution Respirometry: OXPHOS Protocols for Human Cells and Permeabilized Fibers from Small Biopsies of Human Muscle. In: *Methods in molecular biology (Clifton, N.J.)*. 2012, pp 25–58.
5. Lippman R. "An introduction to computing with neural nets." IEEE Trans. ASSP Magazine 4, 4-22, 1987.
6. Hopfield J. "Neural networks and physical systems with emergent collective computational abilities." Proc. Natl. Acad. Sci. (USA) 79, 2554-2558, 1982.
7. Sharmistha P. Chatterjee, Abhijit S. Pandya, Chapter 12 - Artificial Neural Networks in Drug Transport Modeling and Simulation–II, Editor(s): Munish Puri, Yashwant Pathak, Vijay Kumar Sutariya, Srinivas Tipparaju, Wilfrido Moreno, Artificial Neural Network for Drug Design, Delivery and Disposition, Academic Press, 2016, Pages 243-261, ISBN 9780128015599,
8. Jihua Huang, Hiroshi Shimizu, Suteaki Shioya, Gene network Analysis by a Self-Organizing Map and an Artificial Neural Network, IFAC Proceedings Volumes, Volume 37, Issue 3, 2004, Pages 275-280, ISSN 1474-6670,
9. Motalleb G: Artificial neural network analysis in preclinical breast cancer. Cell J. 2014 Winter;15(4):324-31. Epub 2013 Nov 20.

Supplemental Figure 1.

A

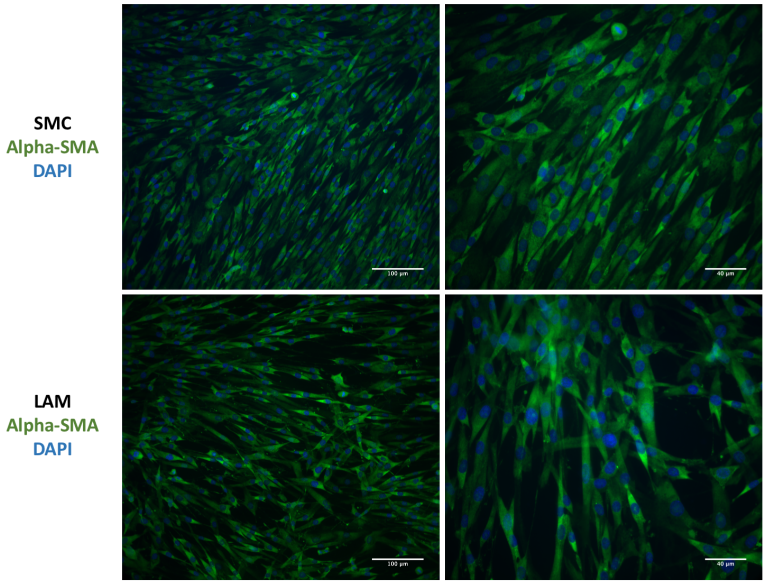

B

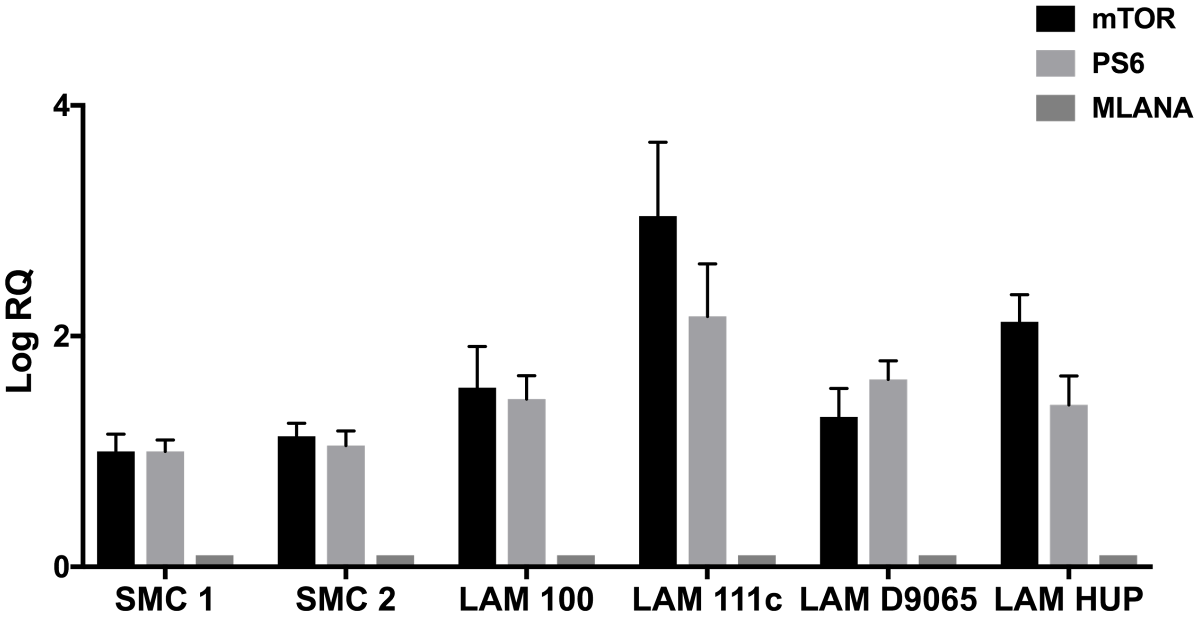

C

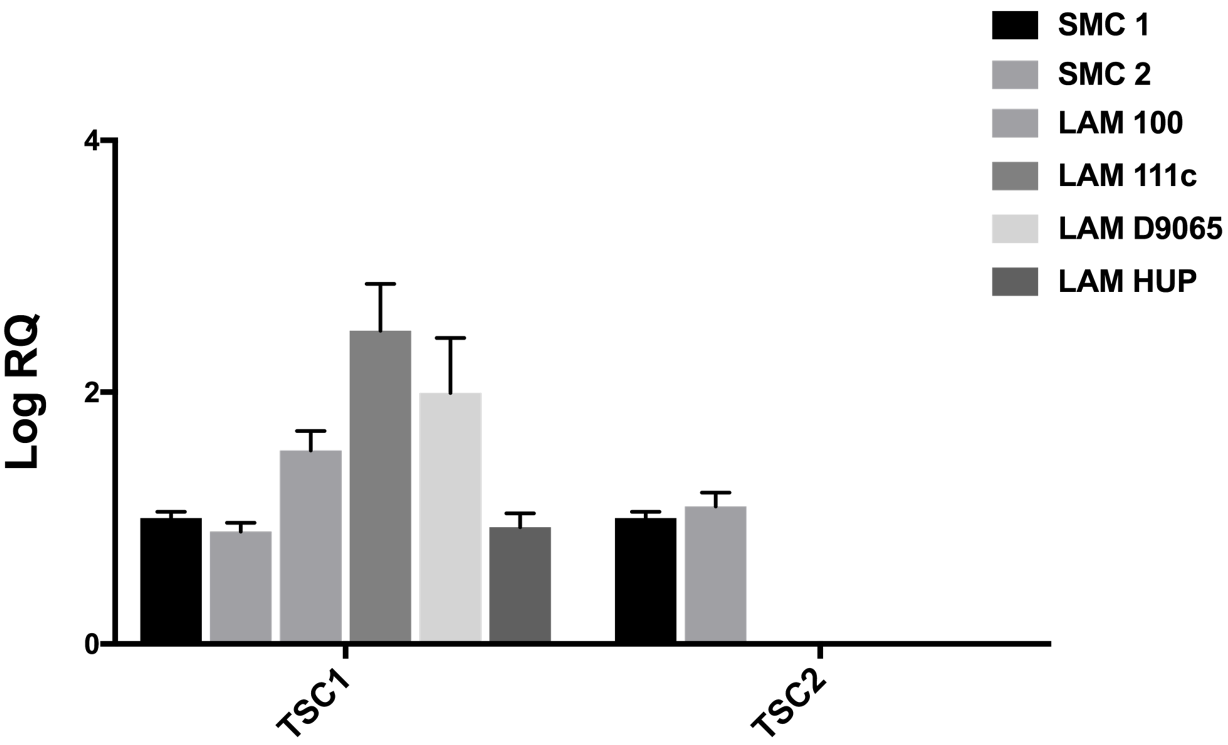

Supplemental Figure 1. Characterization of normal bronchial SMC cells (n=2) and individual patient derived LAM cell lines (n=4). A) ASMA immunofluorescent staining (ASMA green, DAPI blue, objective 20x and 40x, size-bar 100 µm and 40 µm). Each staining is a representative of n=3 technical repeats on SMC (n=2) and LAM (n=4) cell lines each. **B)** qRT-PCR analysis of mTOR, MLANA and PS6 genes. Beta-actin was used as inner control. Data are presented as mean of log RQ ± technical error of the replicates. **C)** qRT-PCR analysis of TSC 1 and 2 genes. Beta-actin was used as inner control, loss of TSC2 gene expression was significant in LAM patient cell lines. Data are presented as mean of log RQ ± technical error of the replicates.

Supplemental Figure 2.

A

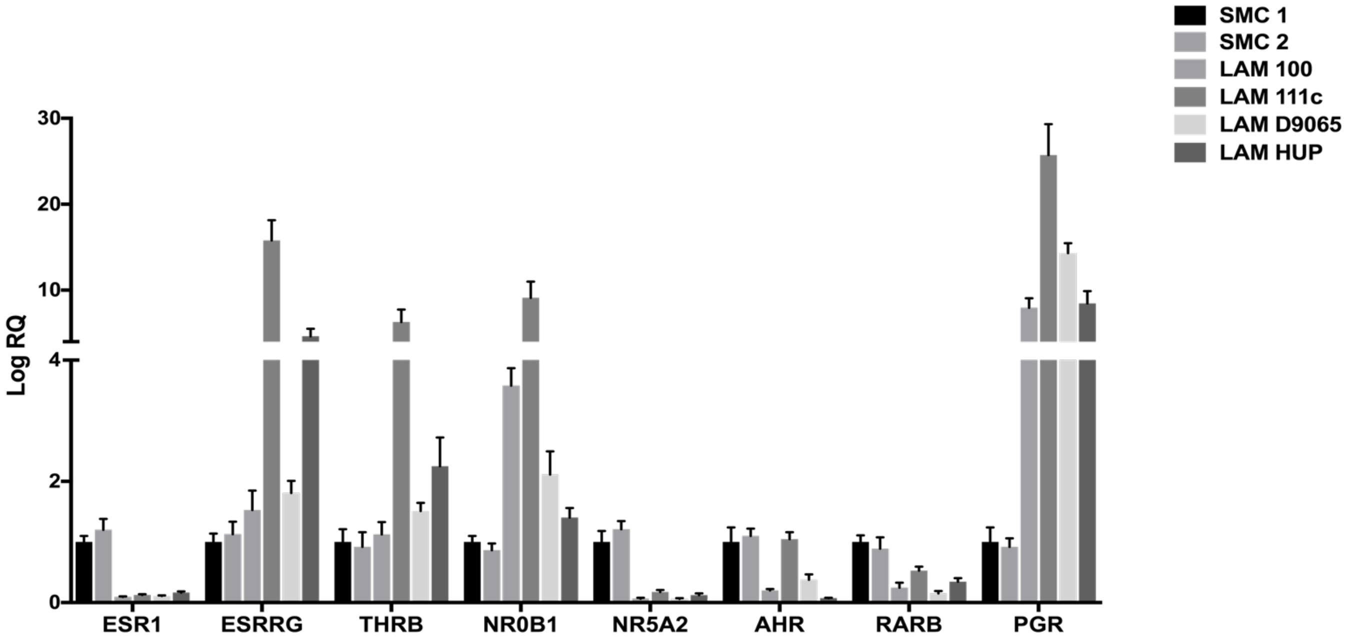

B

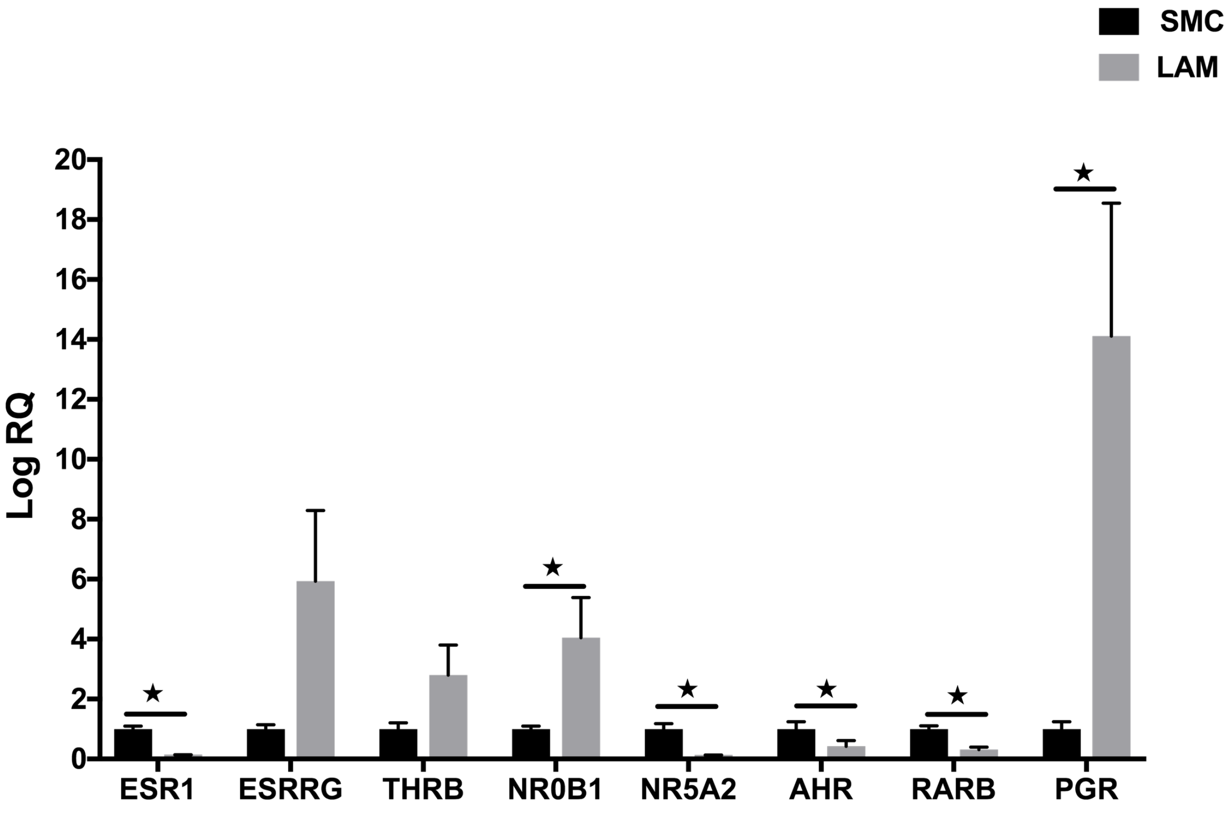

Supplemental Figure 2. qRT-PCR analysis of nuclear receptors on individual LAM samples and normal SMC samples. A) qRT-PCR analysis of ESR1, ESRRG, PGR, THRB, NR0B1, NR5A2, AHR and RARB genes. Beta-actin was used as inner control. Data are presented as mean of log RQ ± technical error of the replicates (SMC n=2). **B)** Data are presented as mean of log RQ±SEM SMC (n=2) and LAM (n=4). Significant changes are marked as ★ ($P<0.05$).

Supplemental figure 3.

A

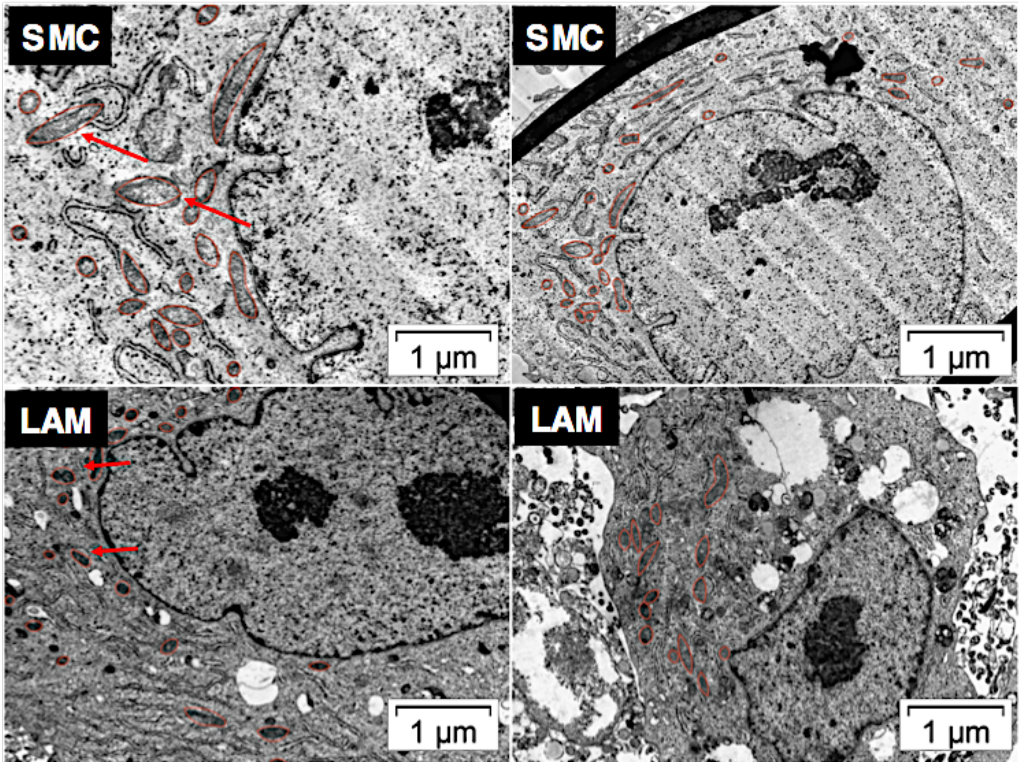

B

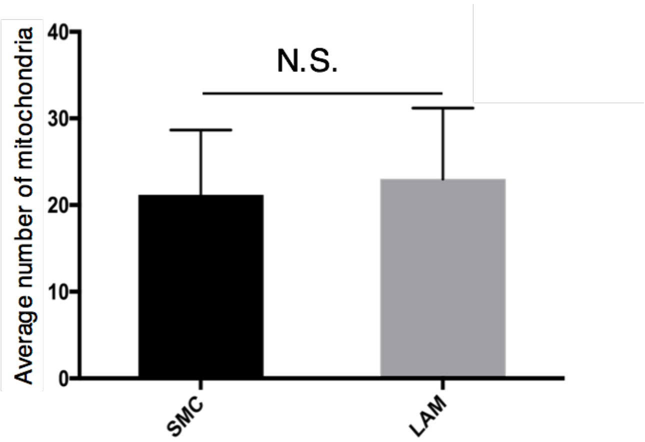

Supplemental Figure 3. **Electron microscopic pictures of a normal SMC control and a LAM cell**.

A) Mitochondria are circled and red arrows point at them out (size bar 1 µm); **B)** Average count of mitochondria in electron microscopic images of normal SMC control (n=2, number of

images=11) and LAM cell types (n=4, number of images=18). Mitochondrial counts were generated using ImageJ. Data are shown as average mitochondria ± SEM.

Supplemental figure 4.

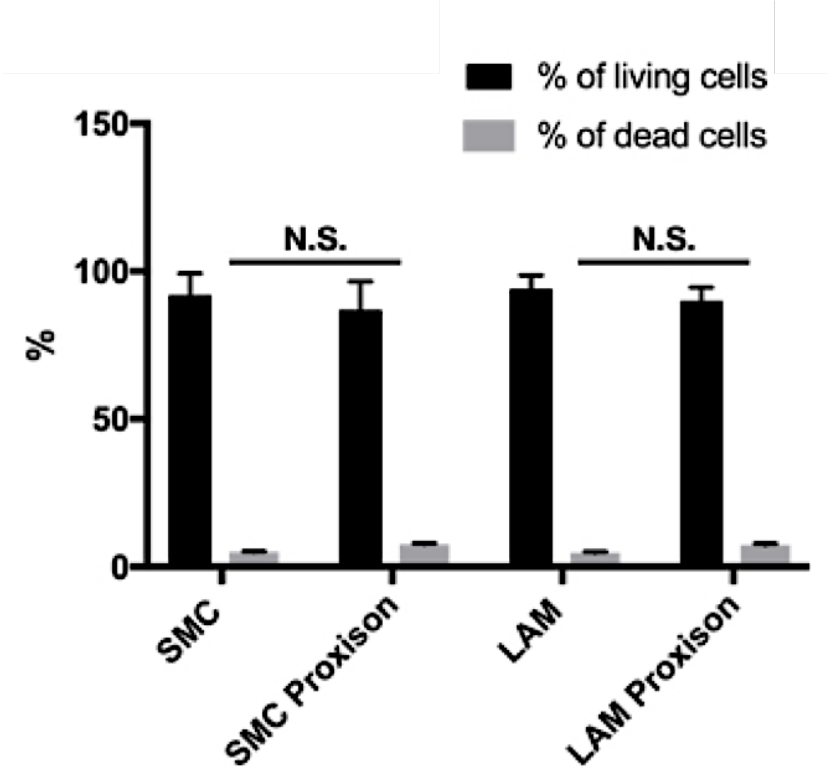

Supplemental Figure 4. **Proxison is not toxic in cell cultures**. After Proxison (3 µM, 1 h) treatment viability of normal SMC (n=2) and LAM cell lines (n=4) were determined using propidium iodide (PI) (500 nM) then fluorescence intensity was analyzed by flow cytometry. Data are presented as percentage of viability in 10,000 cells ± SEM.

Supplemental Figure 5.

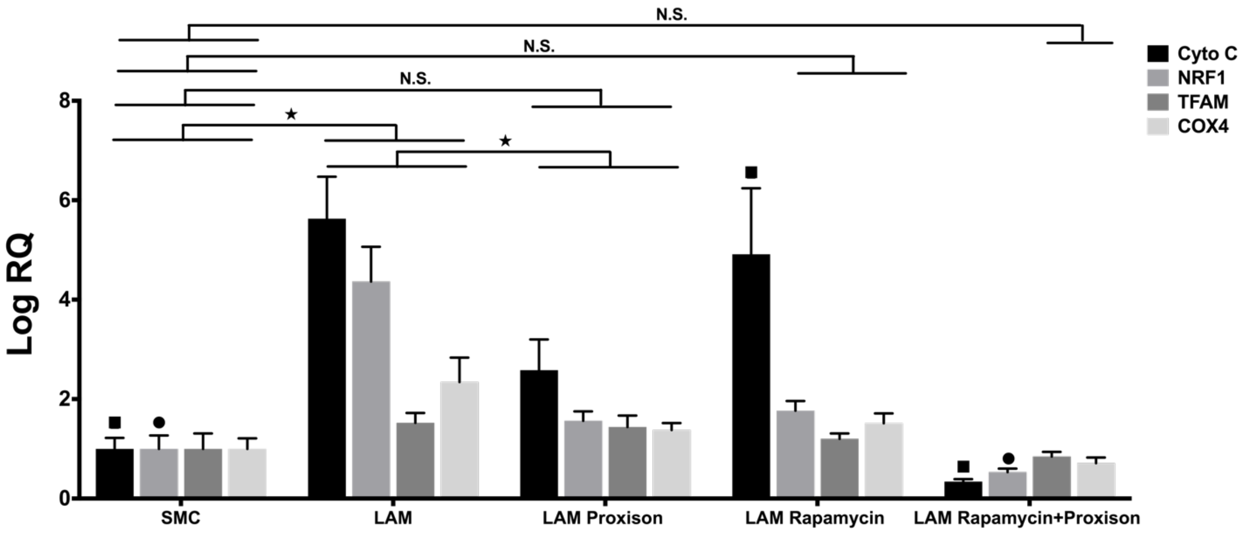

Supplemental Figure 5. **Mitochondrial gene expression changes following Proxison and Rapamycin treatment**. Gene expression in the mitochondria of LAM cell lines (n=4) were compared to normal SMCs (n=2) following Proxison (3 µM, 24h), Rapamycin (20 nM, 24h) and Rapamycin (20 nM, 24h) + Proxison (3 µM, 24h). Data are presented as mean of log RQ ± SEM, significant changes are marked as ★(P<0.05).

Supplemental Figure 6.

A

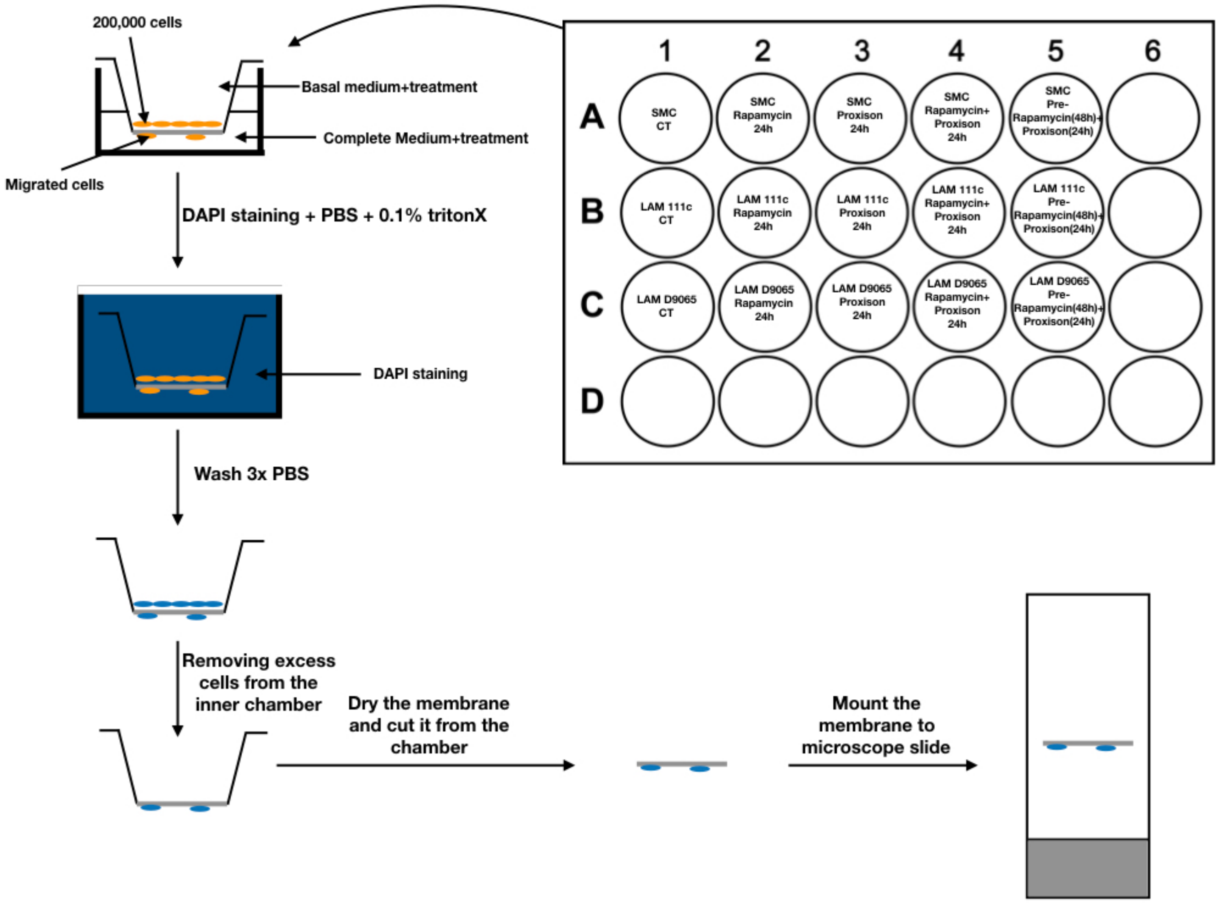

B

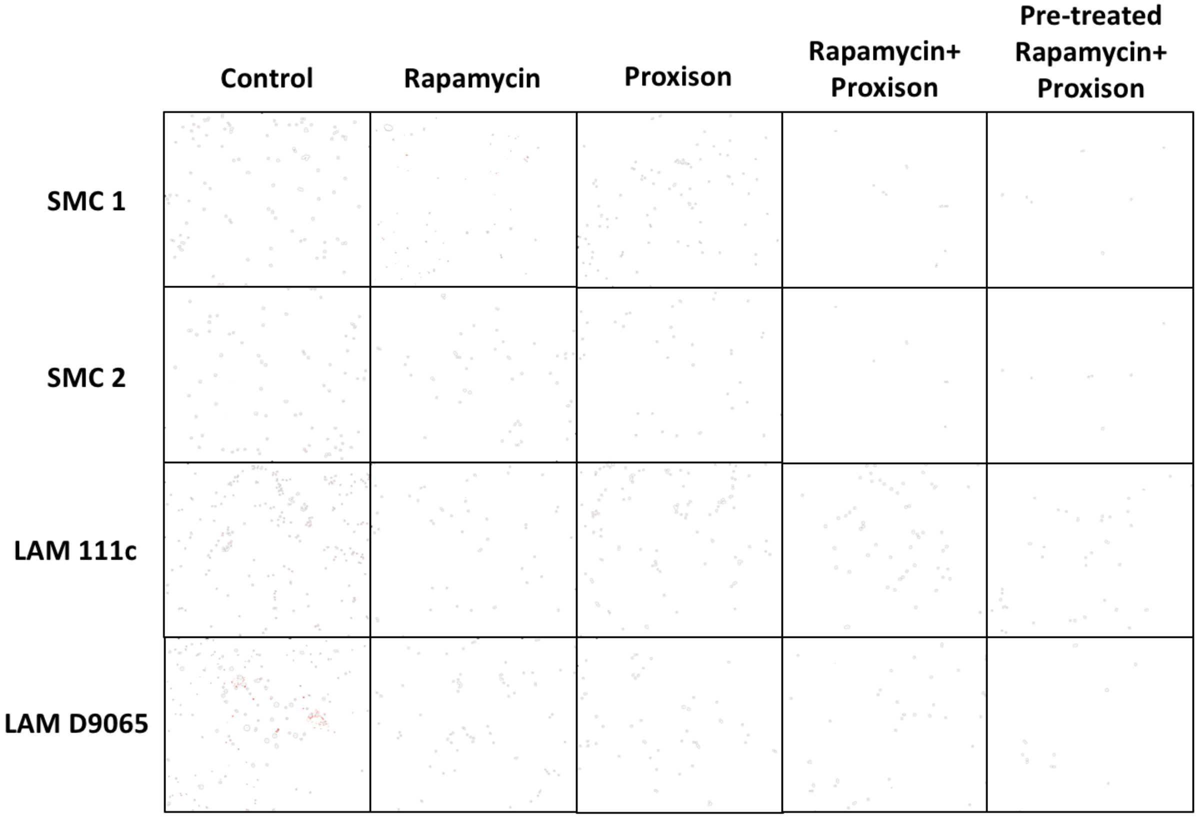
Control
Rapamycin
Proxison
Rapamycin+
Proxison
Pre-treated
Rapamycin+
Proxison
SMC 1
SMC 2
LAM 111c
LAM D9065

C

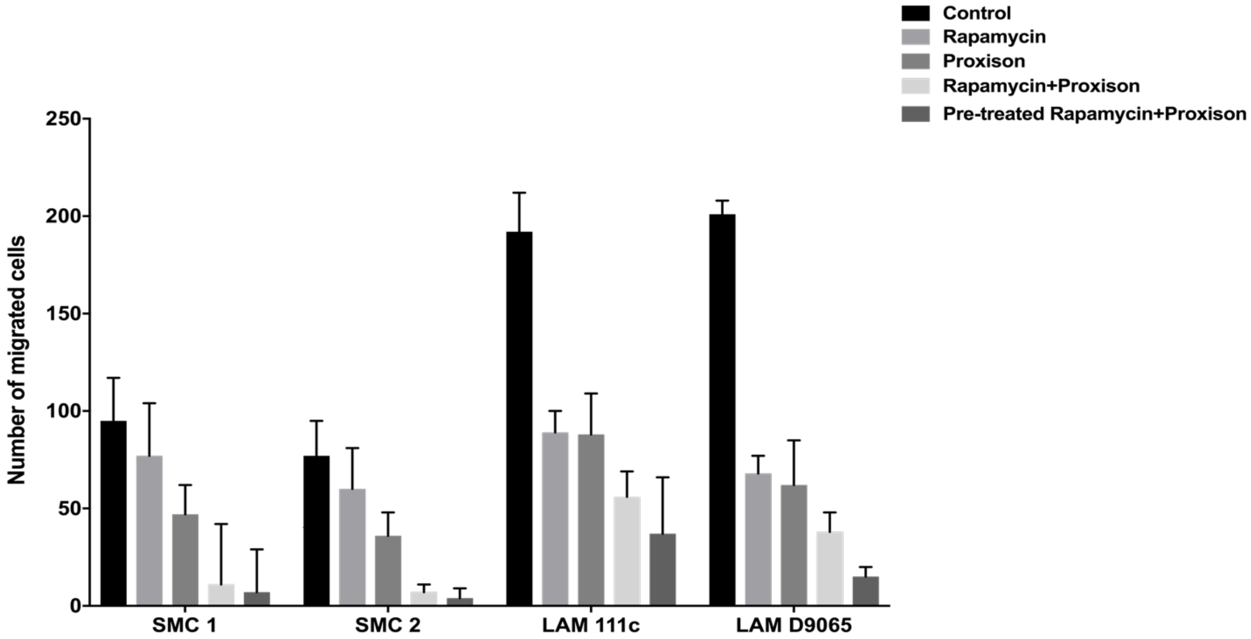
Control
Rapamycin
Proxison
Rapamycin+Proxison
Pre-treated Rapamycin+Proxison
Number of migrated cells
250
200
150
100
50
0
SMC 1
SMC 2
LAM 111c
LAM D9065

Supplemental Figure 6. Migration assay. A) Schematic representation of the migration assay design and treatment strategies. **B)** Migration capacity of LAM cell lines following different treatment strategies Rapamycin (20 nM, 24h), Proxison (3 µM, 24h), Rapamycin (20 nM, 24h) + Proxison (3 µM, 24h) and pre-treated with Rapamycin for 48h (20 nM/24h) + Proxison (3 µM, 24h), images presented as the number of cells migrated through the membrane to the lower side of the chamber and stained with DAPI. **C)** Data are presented as percentage of migrated cells compared to normal SMC ± technical error of the replicates.

GENE	GENE NAME	FOLD CHANGE IN GENE EXPRESSION
**AHR**	**Aryl hydrocarbone receptor**	**0.246833042**
COPS2	COP9 signalosome subunit 2	0.490569164
**ESR1**	**Estrogen Receptor**	**0.248859347**
ESRRB	Estrogen receptor-related receptor beta	0.535669413
**ESRRG**	**Estrogen receptor-related receptor gamma**	**7.969892372**
HDAC2	Histone deacetylase 2	2.014278883
HDAC4	Histone deacetylase 4	0.496454272
HDAC6	Histone deacetylase 6	2.016007928
HDAC7	Histone deacetylase 7	0.495877676
HMGA1	High mobility group AT-hook 1	2.003069563
**HNF4A**	**Hepatocyte nuclear factor alpha**	**0.244270055**
**HNF4G**	**Hepatocyte nuclear factor gamma**	**0.251666716**
KAT5	Lysine acetyltransferase 5	2.041409783
ITGB3BP	Integrin subunit beta 3 binding protein	1.989164659
MED24	Mediator Complex Subunit 24	2.031083036
NCOA1	Nuclear receptor coactivator	0.491716524
NCOA2	Nuclear receptor coactivator	0.497099225
NCOA3	Nuclear receptor coactivator	0.497168236
NCOR1	Nuclear receptor corepressor 1	0.489819992
NOTCH2	notch 2	0.490423258
**NR0B1**	**Nuclear receptor subfamily 0 group B member 1**	**3.985399293**
**NR0B2**	**Nuclear receptor subfamily 0 group B member 2**	**0.249599073**
NR1D1	Nuclear receptor subfamily 1 group D member 1	2.001984093
**NR1H4**	**Nuclear receptor subfamily 1 group H member 4**	**0.219646484**
NR2C2	Nuclear receptor subfamily 2 group C member 2	0.493867798
NR2F6	Nuclear receptor subfamily 2 group F member 6	2.008144646
NR3C2	Nuclear receptor subfamily 3 group C member 2	1.966205721
NR4A1	Nuclear receptor subfamily 4 group A member 1	2.013490782
NR4A2	Nuclear receptor subfamily 4 group A member 2	2.006793749
NR4A3	Nuclear receptor subfamily 4 group A member 3	1.978526651
**NR5A1**	**Nuclear receptor subfamily 5 group A member 1**	**2.66683E-06**
**NR5A2**	**Nuclear receptor subfamily 5 group A member 2**	**0.062379642**
NR6A1	Nuclear receptor subfamily 6 group A member 1	1.988173463
NRIP1	Nuclear receptor interacting protein 1	0.49971055
KAT2B	Lysine acetyltransferase 2B	0.491418875
**PGR**	**Progesterone receptor**	**31.84258578**
PPARA	Peroxisome proliferator-activated receptor alpha	0.498363973
PPARGC1A	Peroxisome proliferator-activated receptor gamma coactivator 1-alpha	0.502162286
**PPARGC1B**	**Peroxisome proliferator-activated receptor gamma coactivator 1-beta**	**7.783457029**
PSMC3	Proteasome 26S subunit ATPase 3	1.997637529
PSMC5	Proteasome 26S subunit ATPase 5	2.012056485
RARA	Retinoic acid receptor alpha	0.494233574
**RARB**	**Retinoic acid receptor beta**	**0.243442787**
RARG	Retinoic acid receptor gamma	0.497948397
RBPJ	Recombining binding protein suppressor of hairless	0.503475858
RORA	RAR related orphan receptor A	0.495202227
**RORC**	**Retinoic acid orphan receptor C**	**11478.81608**
RXRA	Retinoic X receptor alpha	0.494038242
RXRB	Retinoic X receptor beta	0.496204922
RXRG	Retinoic X receptor gamma	2.024292697
**THRB**	**Thyroid hormone receptor beta**	**4.015482279**
VDR	Vitamin D3 receptor	0.49647987

Supplemental Table 2. Nuclear receptors TaqMan array in LAM cells compared to normal SMC control (fold change).

Sensitivity	ESR1-Hs0(	ESRRG-Hs	HNF4A-Hs	HNF4G-Hs	NOTCH2-H	PPARGC1/	PPARGC1I	PGR-Hs00	RARB-Hs0	RXRB-Hs0	RXRG-Hs0	THRB-Hs0	SUM
ESR1-Hs00174860_m1		0.953	0.898	0.934	0.966	1.016	0.848	0.949	0.929	1.049	0.936	0.935	10.413
ESRRG-Hs00155006_m1	1.632		1.735	1.393	1.513	1.421	1.829	1.416	1.433	1.305	1.640	1.714	17.033
HNF4A-Hs00230853_m1	1.026	1.104		0.946	1.044	0.987	1.352	0.962	1.142	0.928	0.964	1.001	11.454
HNF4G-Hs01071345_m1	0.962	1.091	0.892		1.066	0.981	0.820	0.941	1.068	1.132	0.961	1.105	11.020
NOTCH2-Hs01050719_m1	0.495	0.489	0.502	0.478		0.489	0.629	0.486	0.545	0.628	0.482	0.680	5.904
PPARGC1A-Hs01016719_	0.490	0.571	0.460	0.462	0.523		0.794	0.470	0.524	0.426	0.474	0.704	5.898
PPARGC1B-Hs00991677_	1.462	1.387	1.330	1.377	1.474	1.404		1.400	1.344	1.430	1.429	1.361	15.399
PGR-Hs00172183_m1	2.367	2.599	2.553	2.323	2.482	2.394	2.039		2.181	3.645	2.427	2.313	27.323
RARB-Hs00233407_m1	0.939	0.939	0.912	0.948	1.061	1.167	0.905	0.964		0.942	0.965	1.122	10.865
RXRB-Hs00232774_m1	0.537	0.599	0.453	0.470	0.499	0.521	0.423	0.478	0.604		0.477	0.451	5.514
RXRG-Hs00199455_m1	0.478	0.469	0.462	0.473	0.494	0.602	0.431	0.481	0.461	0.461		0.622	5.435
THRB-Hs00230861_m1	0.941	1.145	0.920	0.933	0.966	1.020	1.137	0.949	1.248	1.132	0.984		11.375
AHR-Hs00169233_m1	0.984	1.092	0.917	0.939	0.977	0.973	1.012	0.955	0.834	0.943	1.038	1.060	11.725
AR-Hs00171172_m1	0.006	0.006	0.007	0.006	0.007	0.007	0.006	0.006	0.007	0.010	0.006	0.006	0.080
COPS2-Hs00182826_m1	0.484	0.504	0.463	0.478	0.504	0.507	0.433	0.486	0.840	0.512	0.488	0.572	6.272
CREBBP-Hs00231733_m1	0.004	0.004	0.004	0.004	0.004	0.004	0.004	0.004	0.004	0.004	0.004	0.004	0.046
ESR2-Hs00230957_m1	0.004	0.004	0.004	0.004	0.004	0.004	0.004	0.004	0.004	0.004	0.004	0.004	0.044
ESRRB-Hs01584024_m1	0.456	0.451	0.515	0.419	0.433	0.489	0.506	0.426	0.411	0.424	0.464	0.410	5.405
HDAC2-Hs00231032_m1	0.487	0.510	0.453	0.470	0.491	0.512	0.465	0.478	0.644	0.436	0.478	0.566	5.990
HDAC3-Hs00187320_m1	0.008	0.008	0.008	0.008	0.008	0.008	0.012	0.008	0.008	0.010	0.009	0.010	0.106
HDAC4-Hs01041648_m1	0.516	0.472	0.633	0.470	0.508	0.480	0.510	0.478	0.629	0.559	0.475	0.541	6.271
HDAC5-Hs00608366_m1	0.008	0.009	0.008	0.008	0.009	0.008	0.008	0.008	0.010	0.008	0.008	0.009	0.103
HDAC6-Hs00195869_m1	0.497	0.529	0.488	0.471	0.488	0.491	0.622	0.478	0.478	0.552	0.484	0.500	6.078
HDAC7-Hs00248789_m1	0.476	0.467	0.677	0.471	0.494	0.524	0.436	0.479	0.519	0.476	0.472	0.500	5.991
HMGA1-Hs00852949_g1	0.473	0.477	0.537	0.466	0.483	0.493	0.510	0.474	0.588	0.503	0.508	0.529	6.041
KAT5-Hs00197310_m1	0.485	0.479	0.468	0.479	0.502	0.499	0.654	0.487	0.574	0.772	0.526	0.452	6.376
ITGB3BP-Hs01100612_m1	0.492	0.458	0.637	0.462	0.478	0.475	0.408	0.469	0.581	0.529	0.463	0.479	5.930
MED1-Hs01062349_m1	0.005	0.005	0.005	0.005	0.005	0.005	0.006	0.005	0.004	0.004	0.005	0.005	0.058
MED12-Hs00192801_m1	0.005	0.006	0.005	0.005	0.005	0.005	0.006	0.005	0.006	0.006	0.005	0.006	0.067
MED14-Hs00188481_m1	0.006	0.006	0.006	0.006	0.006	0.006	0.007	0.006	0.007	0.005	0.006	0.006	0.071
MED16-Hs00193899_m1	0.009	0.010	0.012	0.009	0.010	0.010	0.009	0.010	0.009	0.013	0.010	0.010	0.121
MED24-Hs00207863_m1	0.490	0.572	0.473	0.476	0.536	0.513	0.662	0.484	0.444	0.471	0.485	0.632	6.238
MED4-Hs00204327_m1	0.005	0.006	0.005	0.005	0.005	0.005	0.005	0.005	0.004	0.007	0.005	0.005	0.064
NCOA1-Hs00186661_m1	0.497	0.471	0.648	0.476	0.543	0.484	0.421	0.484	0.430	0.472	0.487	0.451	5.866
NCOA2-Hs00896106_m1	0.514	0.471	0.597	0.469	0.494	0.485	0.420	0.477	0.647	0.456	0.485	0.575	6.089
NCOA3-Hs00180722_m1	0.497	0.466	0.462	0.469	0.512	0.493	0.477	0.477	0.421	0.464	0.470	0.580	5.788
NCOR1-Hs00196920_m1	0.512	0.492	0.522	0.479	0.512	0.526	0.474	0.487	0.653	0.468	0.487	0.485	6.097
NONO-Hs00939763_g1	0.005	0.005	0.005	0.005	0.005	0.005	0.005	0.005	0.005	0.004	0.005	0.005	0.058
NR0B1-Hs00230864_m1	1.029	0.980	0.980	0.928	1.014	0.971	1.004	0.944	1.045	0.938	0.956	1.103	11.892
NR0B2-Hs00222677_m1	1.031	0.955	0.981	0.932	0.986	0.976	1.340	0.947	0.979	0.993	1.013	1.073	12.205
NR1D1-Hs00253876_m1	0.496	0.462	0.448	0.466	0.505	0.475	0.423	0.474	0.652	0.502	0.516	0.483	5.902
NR1D2-Hs00233309_m1	0.007	0.007	0.007	0.007	0.008	0.007	0.011	0.007	0.008	0.008	0.007	0.008	0.091
NR1H3-Hs00172885_m1	0.005	0.005	0.005	0.005	0.005	0.005	0.004	0.005	0.004	0.007	0.005	0.006	0.062
NR1H4-Hs00231968_m1	1.013	1.137	1.004	1.017	1.063	1.051	1.089	1.034	1.549	0.964	1.027	1.055	13.005
NR1I2-Hs01114267_m1	0.004	0.005	0.004	0.004	0.004	0.005	0.007	0.004	0.005	0.007	0.004	0.004	0.057
NR2C2-Hs00231489_m1	0.491	0.517	0.456	0.474	0.495	0.483	0.577	0.481	0.430	0.475	0.477	0.452	5.806
NR2E3-Hs00183915_m1	0.019	0.020	0.019	0.018	0.020	0.019	0.018	0.019	0.023	0.019	0.021	0.023	0.236
NR2F1-Hs00818842_m1	0.010	0.009	0.010	0.009	0.009	0.009	0.010	0.009	0.012	0.013	0.009	0.010	0.120
NR2F6-Hs00172870_m1	0.480	0.594	0.526	0.468	0.501	0.484	0.513	0.476	0.509	0.464	0.474	0.484	5.971
NR3C1-Hs00230818_m1	0.006	0.005	0.005	0.005	0.005	0.005	0.008	0.005	0.006	0.005	0.005	0.005	0.065
NR3C2-Hs00230906_m1	0.468	0.494	0.460	0.454	0.472	0.486	0.482	0.461	0.404	0.583	0.473	0.458	5.695
NR4A1-Hs00374230_m1	0.509	0.465	0.462	0.470	0.491	0.478	0.512	0.478	0.484	0.794	0.502	0.476	6.120
NR4A2-Hs00428691_m1	0.466	0.551	0.454	0.468	0.486	0.476	0.423	0.475	0.604	0.468	0.472	0.812	6.155
NR4A3-Hs00545009_g1	0.476	0.471	0.672	0.458	0.503	0.468	0.445	0.466	0.519	0.482	0.468	0.494	5.920
NR5A2-Hs00187067_m1	2.189	2.069	1.956	1.862	1.981	1.898	2.363	1.893	1.614	3.171	1.887	2.508	25.391
NR6A1-Hs00364256_m1	0.483	0.576	0.544	0.461	0.510	0.471	0.446	0.469	0.484	0.505	0.465	0.454	5.868
NRIP1-Hs00942766_s1	0.462	0.467	0.537	0.466	0.498	0.566	0.584	0.473	0.669	0.449	0.467	0.439	6.078
KAT2B-Hs00187332_m1	0.472	0.498	0.673	0.477	0.501	0.526	0.674	0.485	0.517	0.447	0.498	0.536	6.303
PPARA-Hs00231882_m1	0.471	0.601	0.664	0.467	0.511	0.477	0.474	0.475	0.421	0.681	0.529	0.489	6.261
PPARD-Hs00602622_m1	0.004	0.004	0.004	0.004	0.004	0.004	0.004	0.004	0.005	0.004	0.004	0.004	0.050
PPARG-Hs00234592_m1	0.004	0.004	0.004	0.004	0.004	0.004	0.006	0.004	0.004	0.004	0.004	0.004	0.050
PSMC3-Hs00267682_m1	0.476	0.487	0.468	0.464	0.482	0.494	0.685	0.472	0.462	0.545	0.468	0.440	5.946
PSMC5-Hs00267687_m1	0.492	0.494	0.532	0.469	0.489	0.487	0.499	0.477	0.668	0.586	0.472	0.543	6.208
RARA-Hs00940446_m1	0.518	0.471	0.456	0.473	0.494	0.482	0.606	0.481	0.537	0.532	0.486	0.499	6.035
RARG-Hs00171273_m1	0.518	0.479	0.499	0.468	0.488	0.482	0.439	0.476	0.430	0.436	0.479	0.600	5.794
RBPJ-Hs00794653_m1	0.475	0.485	0.502	0.461	0.480	0.529	0.471	0.468	0.532	0.433	0.506	0.664	6.004
RORA-Hs00536545_m1	0.463	0.467	0.471	0.472	0.489	0.502	0.854	0.480	0.479	0.506	0.473	0.476	6.133
RORB-Hs00199445_m1	0.007	0.007	0.006	0.007	0.007	0.007	0.008	0.007	0.006	0.008	0.007	0.006	0.084
RXRA-Hs00172565_m1	0.478	0.584	0.593	0.473	0.517	0.482	0.748	0.481	0.903	0.439	0.496	0.731	6.926
TGS1-Hs00227358_m1	0.004	0.004	0.005	0.004	0.004	0.004	0.004	0.004	0.006	0.004	0.004	0.004	0.051
THRA-Hs00268470_m1	0.004	0.004	0.004	0.004	0.004	0.004	0.006	0.004	0.006	0.004	0.004	0.004	0.051
TRIP4-Hs00249373_m1	0.005	0.004	0.004	0.004	0.004	0.004	0.007	0.004	0.005	0.004	0.005	0.005	0.055
VDR-Hs01045840_m1	0.486	0.483	0.531	0.470	0.486	0.478	0.414	0.478	0.520	0.512	0.478	0.524	5.860
SUM	33.773	34.192	34.622	31.991	34.630	34.314	35.447	31.102	35.741	37.183	33.780	36.285	

Sensitivity color code	0 - 0.500	).501 - 1.000	.001 - 2.000	.001 - 3.00(	> 3.001

Supplemental Table 3. ANN analysis of nuclear receptors array.

	SMC	LAM
Activin A	4265	4887.666667
ADAMTS-1	1399.5	1711.333333
Angiogenin	1704.5	2322.833333
Angiopoietin-1	4188	5308.5
Angiopoietin-2	9787	9318.5
Angiostatin/Plasminogen	2840	3235.5
Amphiregulin	2454	3210.666667
Artemin	5750.5	5442.833333
**Coagulation Factor III**	**3115**	**15816.5**
**CXCL16**	**2082.5**	**14733.83333**
DPPIV	9097.5	9576.333333
EGF	1107	1086.666667
EG-VEGF	3492.5	3075
**Endoglin**	**2048**	**3380.833333**
Endostatin/Collagen XVIII	2335.5	2073.333333
**Endothelin-1**	**13332.5**	**11469.33333**
FGF acidic	3620	2801
**FGF basic**	**2823**	**13145.5**
FGF-4	1629.5	1600.666667
**FGF-7**	**572**	**1052.666667**
GDNF	2005.5	1880.833333
GM-CSF	1893	1675.166667
HB-EGF	2784	2564.5
HGF	2204	2232.333333
IGFBP-1	6578	5212.833333
IGFBP-2	1782	1546
IGFBP-3	3908	3677.333333
IL-1β	2013.5	1684
LAP (TGF-β1)	2127	2526.5
Leptin	1014.5	1569.166667
**MCP-1**	**752**	**2519.166667**
MIP-1α	3519	3029.666667
MMP-8	2489.5	2002.75
MMP-9	2986.5	2589.666667
NRG1-β1	2037	1778
Pentraxin 3 (PTX3)	5368.5	4717.5
PD-ECGF	2957	2394.166667
PDGF-AA	2638	2177.666667
PDGF-AB/PDGF-BB	1036.5	953.5
Persephin	7634.5	6489.5
Platelet Factor 4 (PF4)	2442	2524.5
**PIGF**	**2538.5**	**3752.5**
Prolactin	1949.5	2396
Serpin B5	2860.5	2933.5
**Serpin E1**	**15933.5**	**1834**
Serpin F1	5260	4779.5
**TIMP-1**	**7144.5**	**13233.5**
TIMP-4	3462	2918.166667
Thrombospondin-1	21223.5	18811
Thrombospondin-2	4154.5	3729.166667
**uPA**	**6238**	**13104**
Vasohibin	4284	3879
VEGF	2054.5	2476.166667
**VEGF-C**	**1478**	**2595**
**IL-8**	**1658.5**	**4653.333333**

Supplemental Table 4. Angiogenesis protein array. Data is presented as mean of pixel intensity in both normal SMC control and LAM cells.

Sensitivity	Endothelin	FGF basic	IL-8	MCP-1	TIMP-1	VEGF-C	SUM
Endothelin-1		846.435	959.987	816.482	853.177	846.132	4322.213
FGF basic	4956.650		4715.427	4988.697	4773.568	4665.403	24099.745
IL-8	1299.729	1187.348		1390.009	1358.681	1341.215	6576.982
MCP-1	1087.942	691.797	687.180		825.387	806.940	4099.246
TIMP-1	2612.796	2656.639	2848.314	2820.399		2920.958	13859.107
VEGF-C	479.514	553.909	536.589	482.343	507.294		2559.647
Activin A	266.300	288.171	231.391	262.720	291.212	283.845	1623.639
ADAMTS-1	144.877	115.017	140.880	143.971	142.806	156.087	843.637
Angiogenin	268.060	424.238	258.012	298.918	280.562	275.977	1805.767
Angiopoietin-1	492.178	811.680	474.231	556.263	535.910	516.315	3386.576
Angiopoietin-2	259.490	196.789	191.784	214.185	233.432	204.234	1299.915
Angiostatin/Plasminogen	196.760	147.149	156.631	166.809	184.647	172.930	1024.925
Amphiregulin	344.225	294.554	322.181	348.522	346.554	348.780	2004.816
Artemin	138.666	129.351	131.529	132.501	139.593	151.503	823.141
Coagulation Factor III	7988.118	5016.408	5562.977	5394.201	5818.673	5896.713	35677.090
CXCL16	5465.105	8193.719	5053.841	6199.150	5861.603	5952.780	36726.198
DPPIV	204.874	282.486	212.422	227.183	232.306	206.003	1365.274
EG-VEGF	180.953	204.033	165.295	223.586	196.010	209.415	1179.291
Endoglin	624.338	772.439	547.943	578.656	605.022	584.922	3713.319
Endostatin/Collagen XVIII	133.706	112.406	159.498	109.278	119.923	134.343	769.155
FGF acidic	350.786	291.371	345.859	357.283	374.741	363.230	2083.270
FGF-7	228.111	196.415	178.740	220.801	236.202	221.594	1281.864
IGFBP-1	583.402	566.470	500.395	747.859	622.490	618.988	3639.604
IL-1β	150.226	171.739	134.171	192.256	153.653	161.024	963.069
LAP (TGF-β1)	182.990	227.550	156.306	207.912	185.079	174.443	1134.280
Leptin	251.406	283.293	221.453	262.507	261.437	252.383	1532.480
MIP-1α	213.201	183.990	205.949	256.104	225.388	224.326	1308.958
MMP-8	211.509	196.413	193.463	227.566	220.890	221.062	1270.903
MMP-9	221.387	149.208	220.221	182.451	181.552	173.100	1127.920
NRG1-β1	116.159	115.018	125.129	117.229	118.780	114.817	707.132
Pentraxin 3 (PTX3)	287.223	262.585	302.465	292.743	322.450	309.709	1777.174
PD-ECGF	292.156	249.161	250.816	258.880	264.239	261.859	1577.111
PDGF-AA	196.849	209.174	191.443	202.989	225.201	261.143	1286.800
Persephin	489.160	492.910	453.478	544.794	528.822	499.047	3008.211
PIGF	689.377	454.724	671.887	543.113	562.751	583.094	3504.945
Prolactin	195.335	192.353	182.520	216.285	211.527	244.157	1242.177
Serpin E1	9113.180	5594.563	5402.603	6239.449	6571.685	6511.436	39432.917
Serpin F1	206.274	182.903	239.225	211.621	218.242	227.764	1286.028
TIMP-4	249.392	228.117	249.566	245.664	264.707	253.996	1491.442
Thrombospondin-1	1126.451	1168.525	1042.923	1135.291	1094.995	1195.648	6763.833
Thrombospondin-2	196.404	173.760	163.017	227.644	196.801	187.176	1144.803
uPA	2963.300	2789.770	3727.029	2914.810	3199.771	3043.316	18637.996
Vasohibin	177.586	191.335	151.472	210.629	183.726	191.951	1106.699
VEGF	184.804	190.282	181.257	194.019	191.722	183.180	1125.265
SUM	46020.948	37686.196	38847.500	41563.772	39923.209	42152.939	

	0 - 500	501 - 2000	2001 - 5000	5001 - 8000	> 8000
**Sensitivity color code**					

Supplemental Table 5. ANN analysis of Angiogenesis protein array.
