# Supplementary material for: Mitochondrial dysfunction is a key determinant of the rare disease lymphangioleiomyomatosis and provides a novel therapeutic target
Source: Oncogene. 2018 Dec 20;38(16):3093–101. doi: 10.1038/s41388-018-0625-1 (PMC6484686; doi:10.1038/s41388-018-0625-1)
Supplement: Supplementary file 2 — Individual data points [file 41388_2018_625_MOESM2_ESM.pdf]

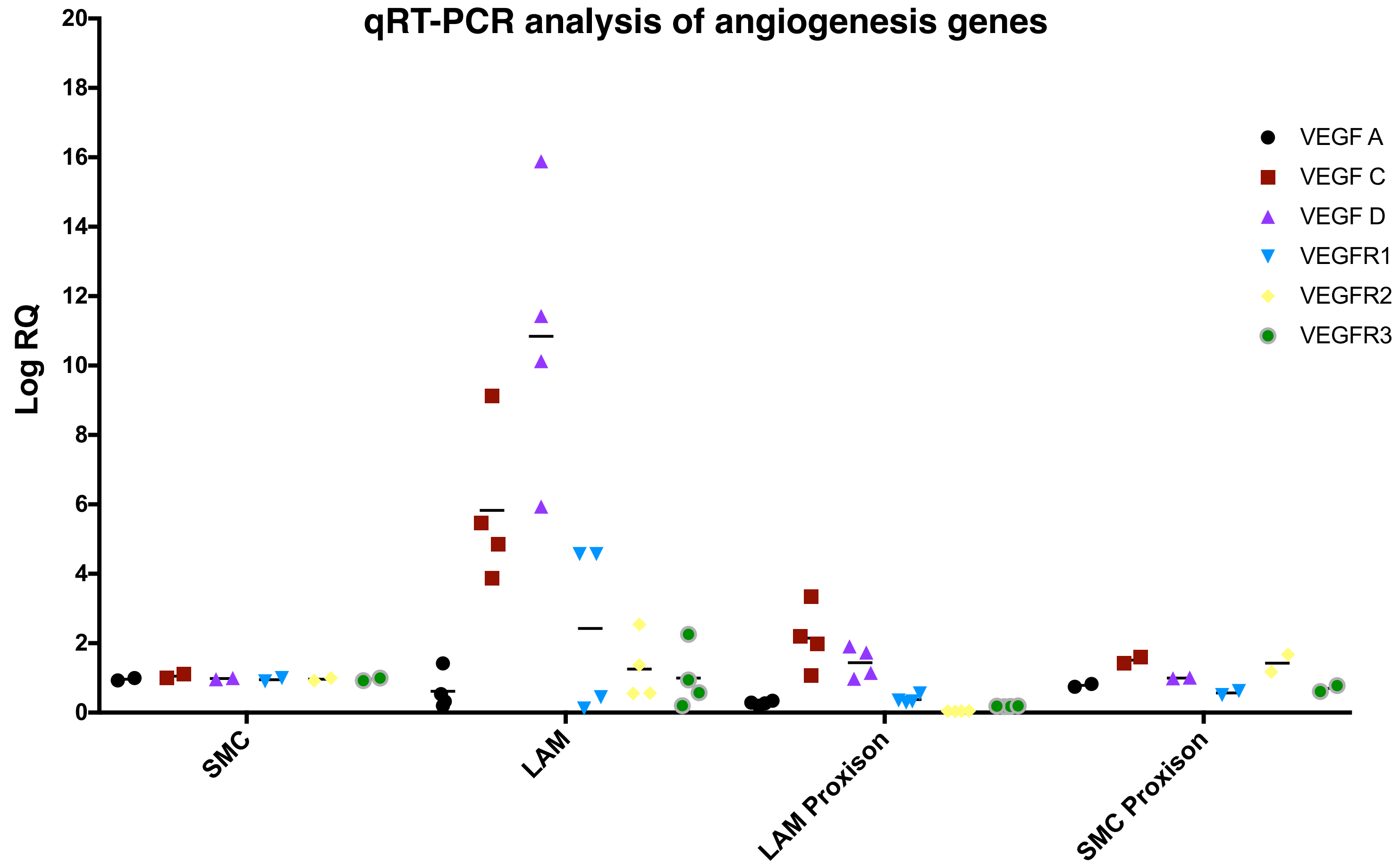

qRT-PCR analysis of angiogenesis genes
Log RQ
0
2
4
6
8
10
12
14
16
18
20
SMC
LAM
LAM Proxison
SMC Proxison
VEGF A
VEGF C
VEGF D
VEGFR1
VEGFR2
VEGFR3

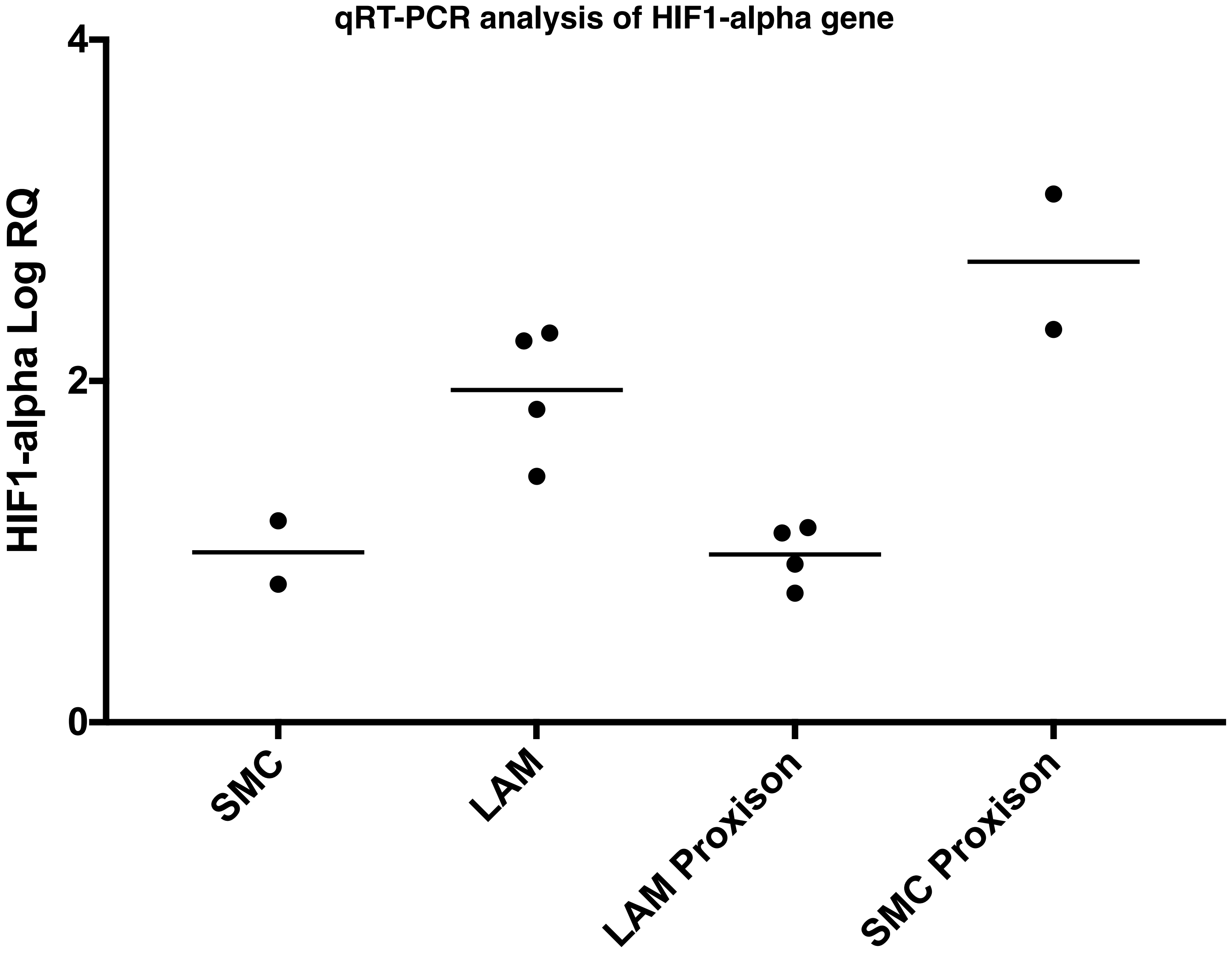

qRT-PCR analysis of HIF1-alpha gene
HIF1-alpha Log RQ
4
2
0
SMC
LAM
LAM Proxison
SMC Proxison

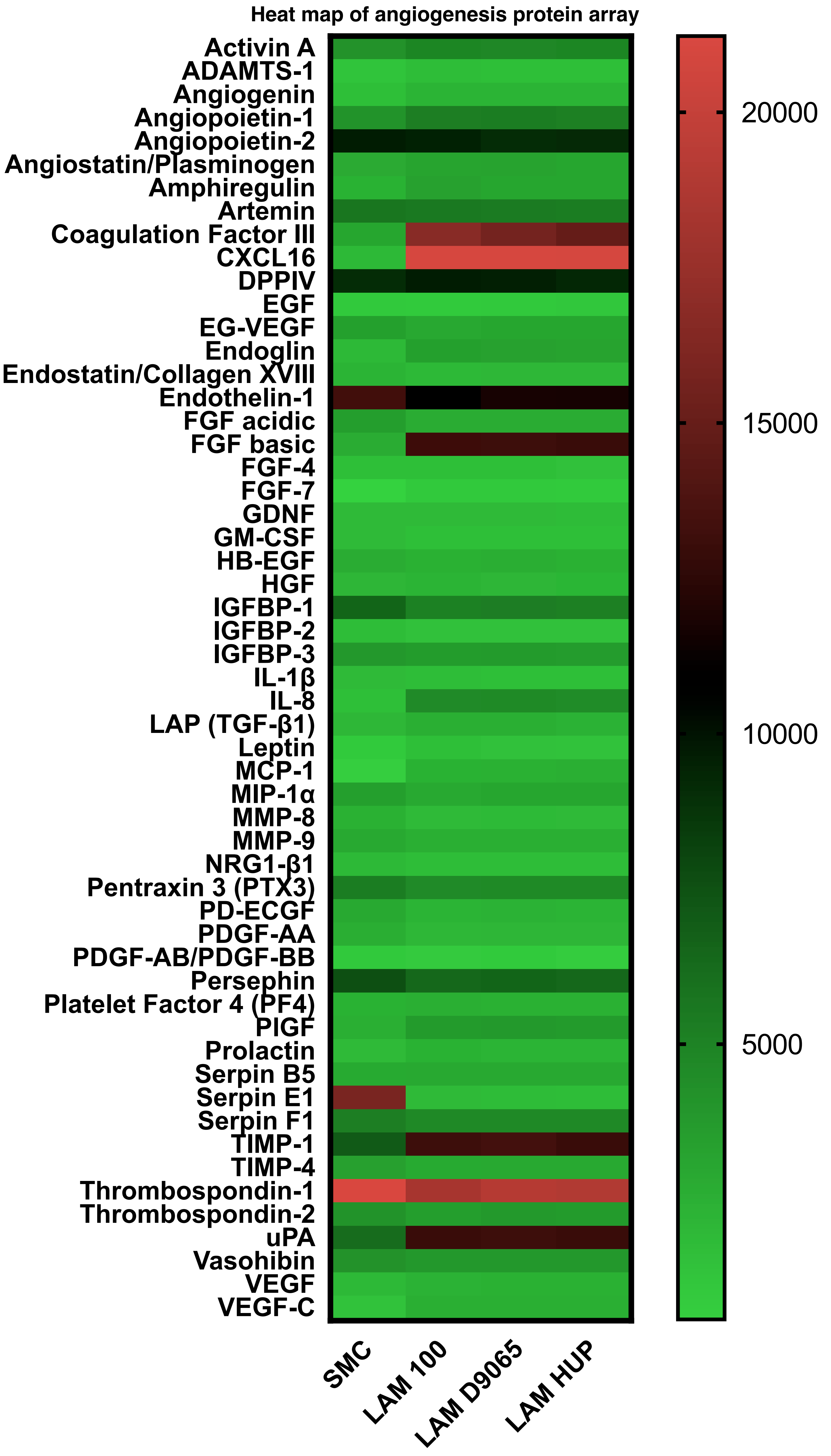
Heat map of angiogenesis protein array
Activin A
ADAMTS-1
Angiogenin
Angiopoietin-1
Angiopoietin-2
Angiostatin/Plasminogen
Amphiregulin
Artemin
Coagulation Factor III
CXCL16
DPPIV
EGF
EG-VEGF
Endoglin
Endostatin/Collagen XVIII
Endothelin-1
FGF acidic
FGF basic
FGF-4
FGF-7
GDNF
GM-CSF
HB-EGF
HGF
IGFBP-1
IGFBP-2
IGFBP-3
IL-1β
IL-8
LAP (TGF-β1)
Leptin
MCP-1
MIP-1α
MMP-8
MMP-9
NRG1-β1
Pentraxin 3 (PTX3)
PD-ECGF
PDGF-AA
PDGF-AB/PDGF-BB
Persephin
Platelet Factor 4 (PF4)
PIGF
Prolactin
Serpin B5
Serpin E1
Serpin F1
TIMP-1
TIMP-4
Thrombospondin-1
Thrombospondin-2
uPA
Vasohibin
VEGF
VEGF-C
SMC
LAM 100
LAM D9065
LAM HUP
20000
15000
10000
5000

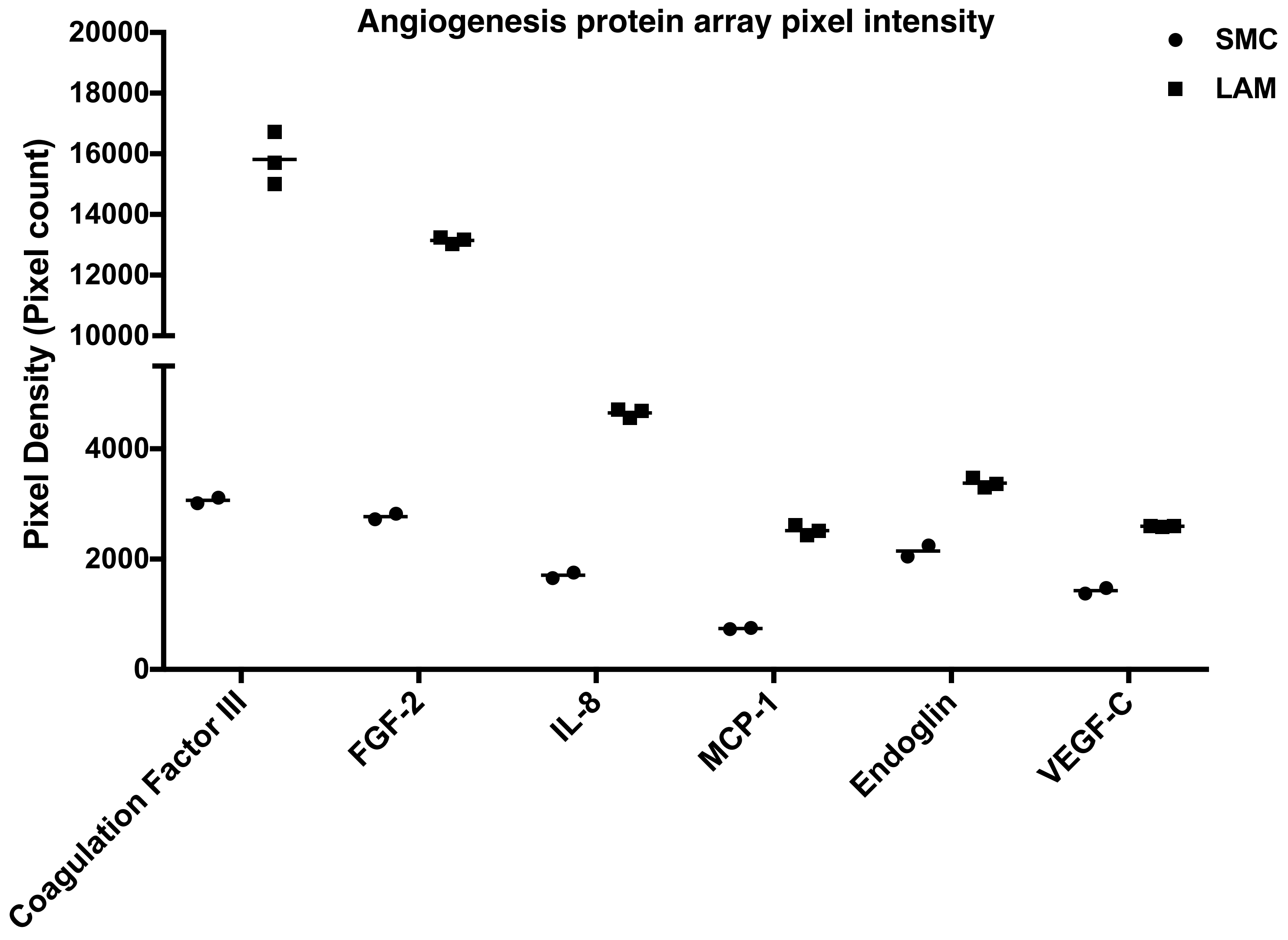

Angiogenesis protein array pixel intensity
SMC
LAM
Pixel Density (Pixel count)
20000
18000
16000
14000
12000
10000
4000
2000
0
Coagulation Factor III
FGF-2
IL-8
MCP-1
Endoglin
VEGF-C

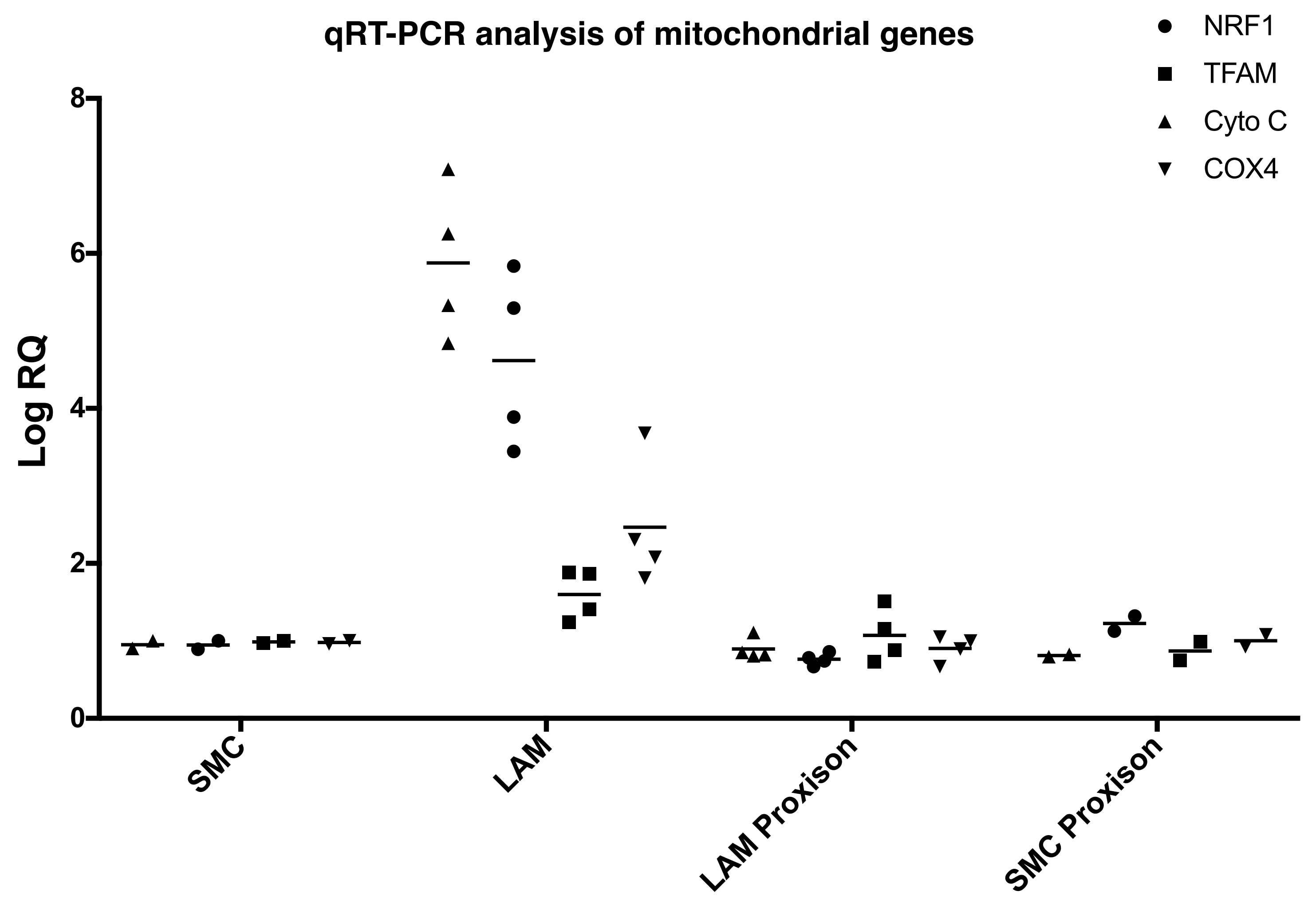
qRT-PCR analysis of mitochondrial genes
NRF1
TFAM
Cyto C
COX4
Log RQ
8
6
4
2
0
SMC
LAM
LAM Proxison
SMC Proxison

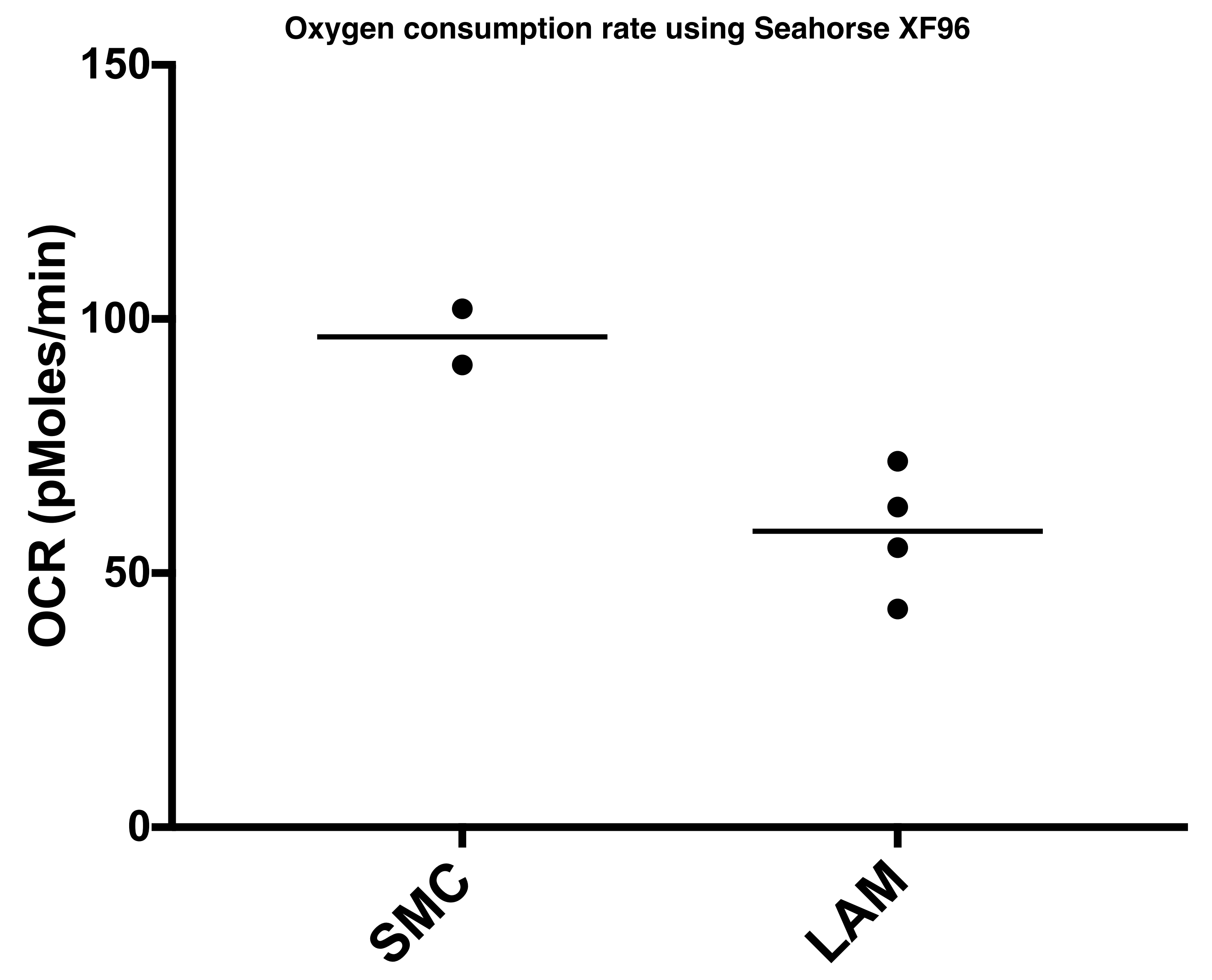
Oxygen consumption rate using Seahorse XF96
OCR (pMoles/min)
150
100
50
0
SMC
LAM

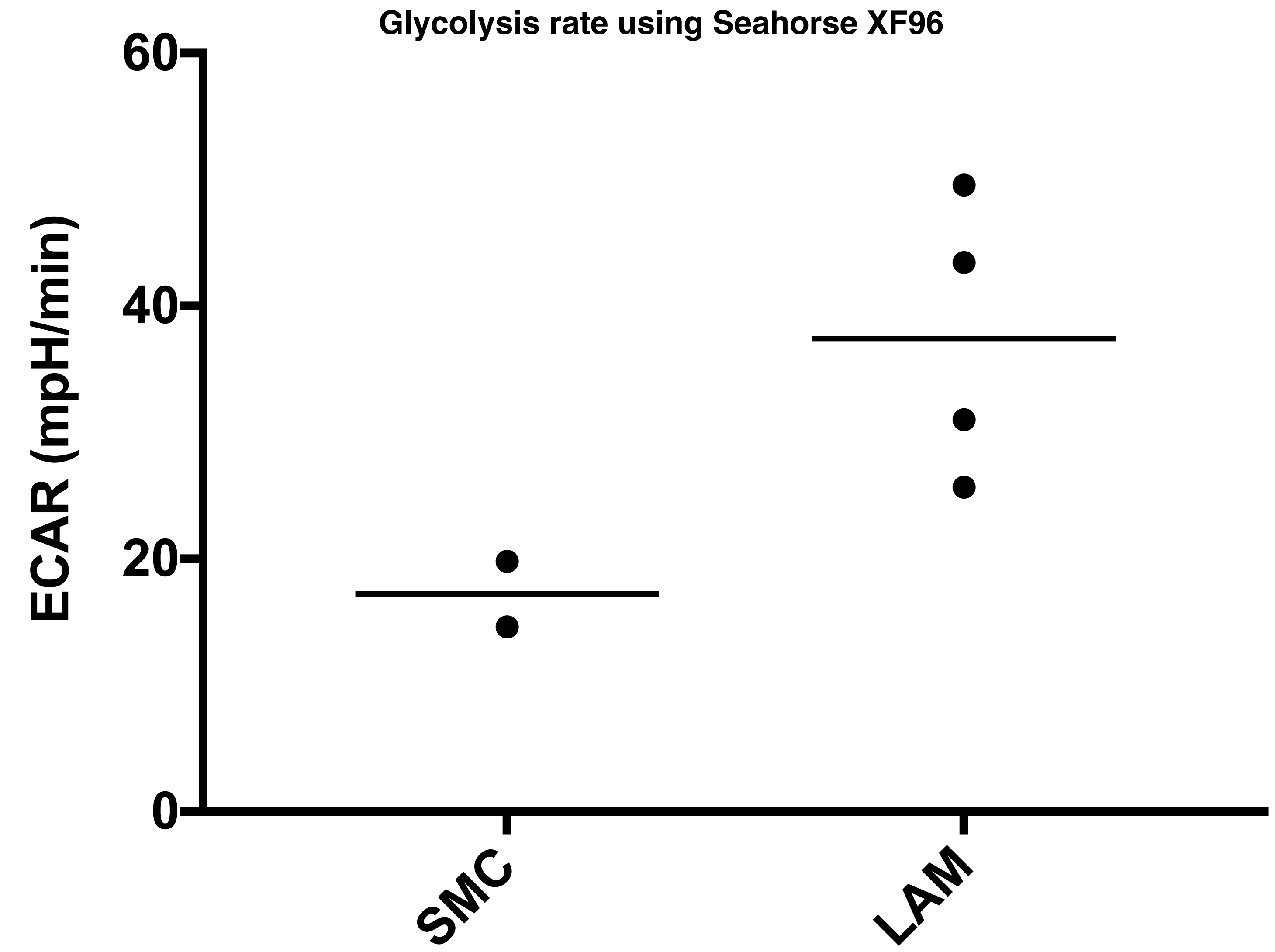

Glycolysis rate using Seahorse XF96
ECAR (mpH/min)
60
40
20
0
SMC
LAM

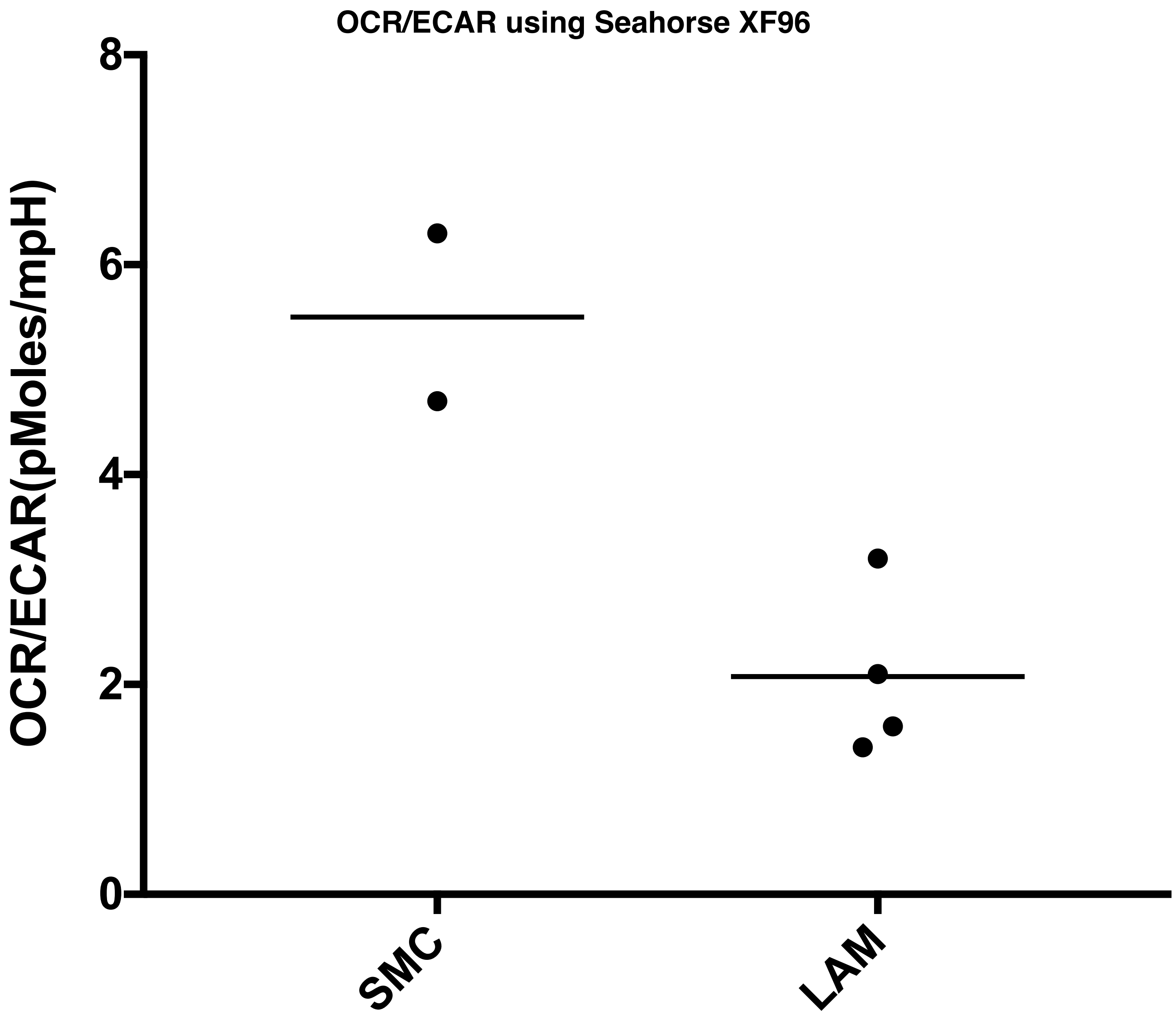
OCR/ECAR using Seahorse XF96
OCR/ECAR(pMoles/mpH)
8
6
4
2
0
SMC
LAM

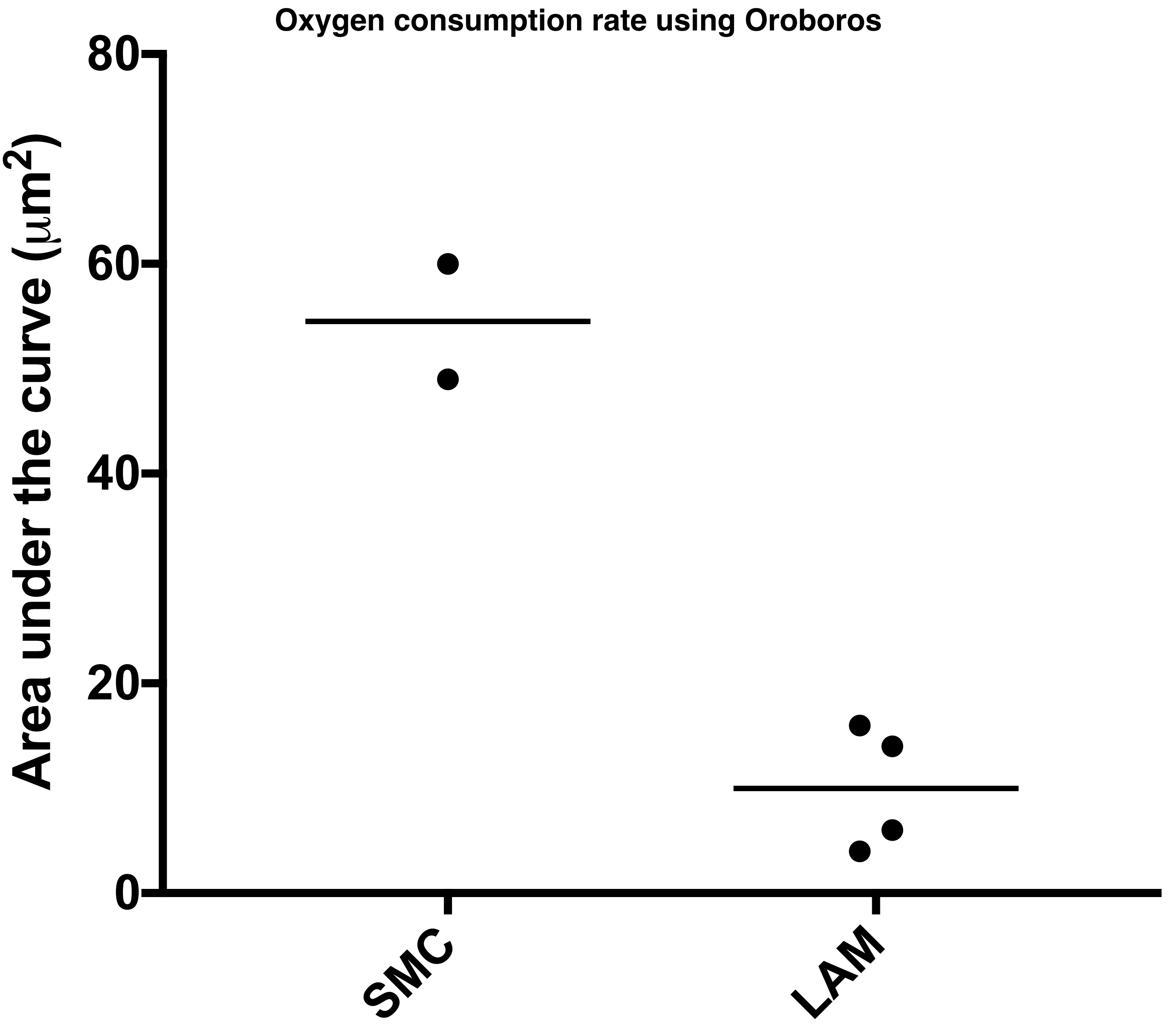
Oxygen consumption rate using Oroboros
Area under the curve (μm2)
80
60
40
20
0
SMC
LAM

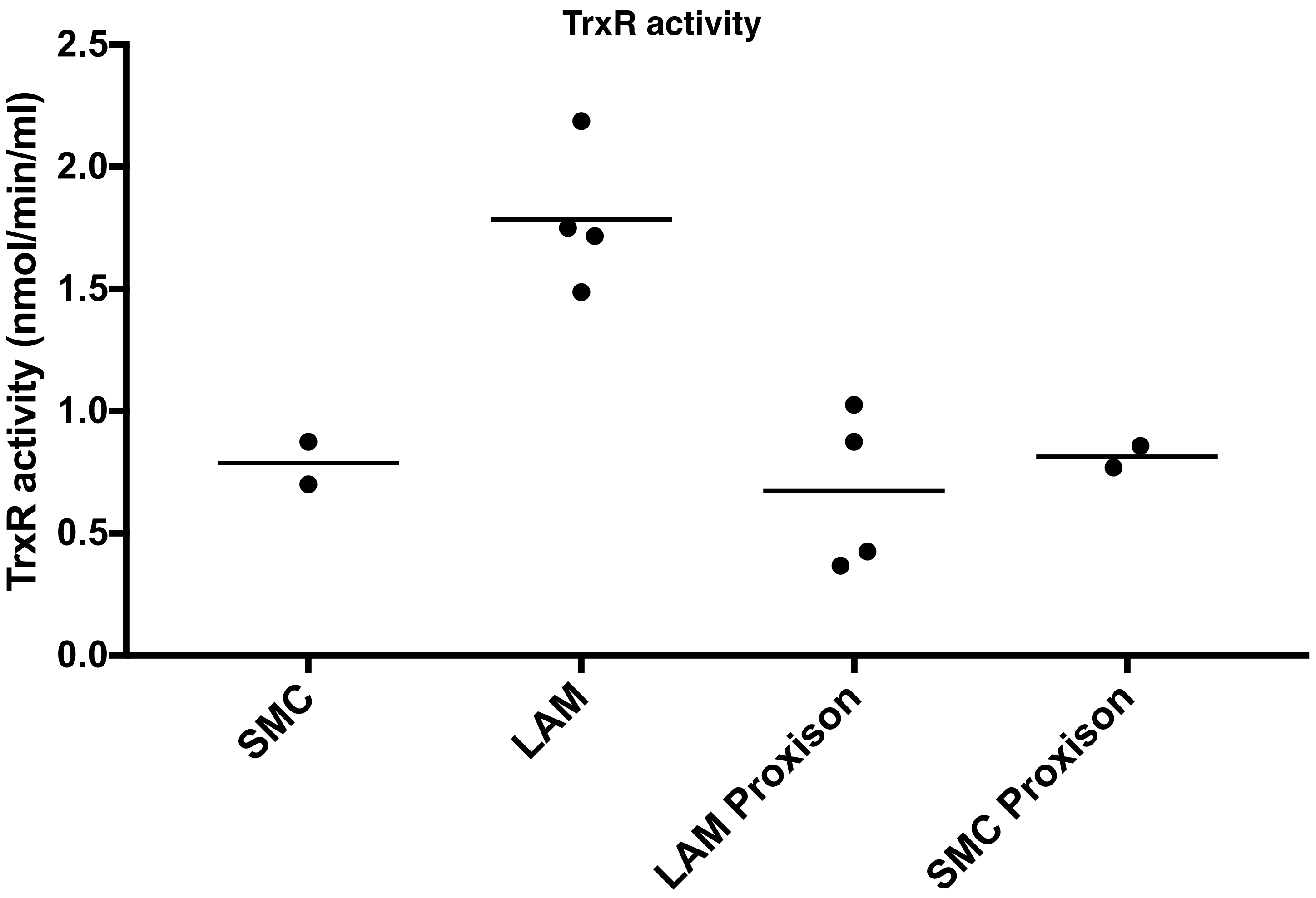
TrxR activity
TrxR activity (nmol/min/ml)
2.5
2.0
1.5
1.0
0.5
0.0
SMC
LAM
LAM Proxison
SMC Proxison

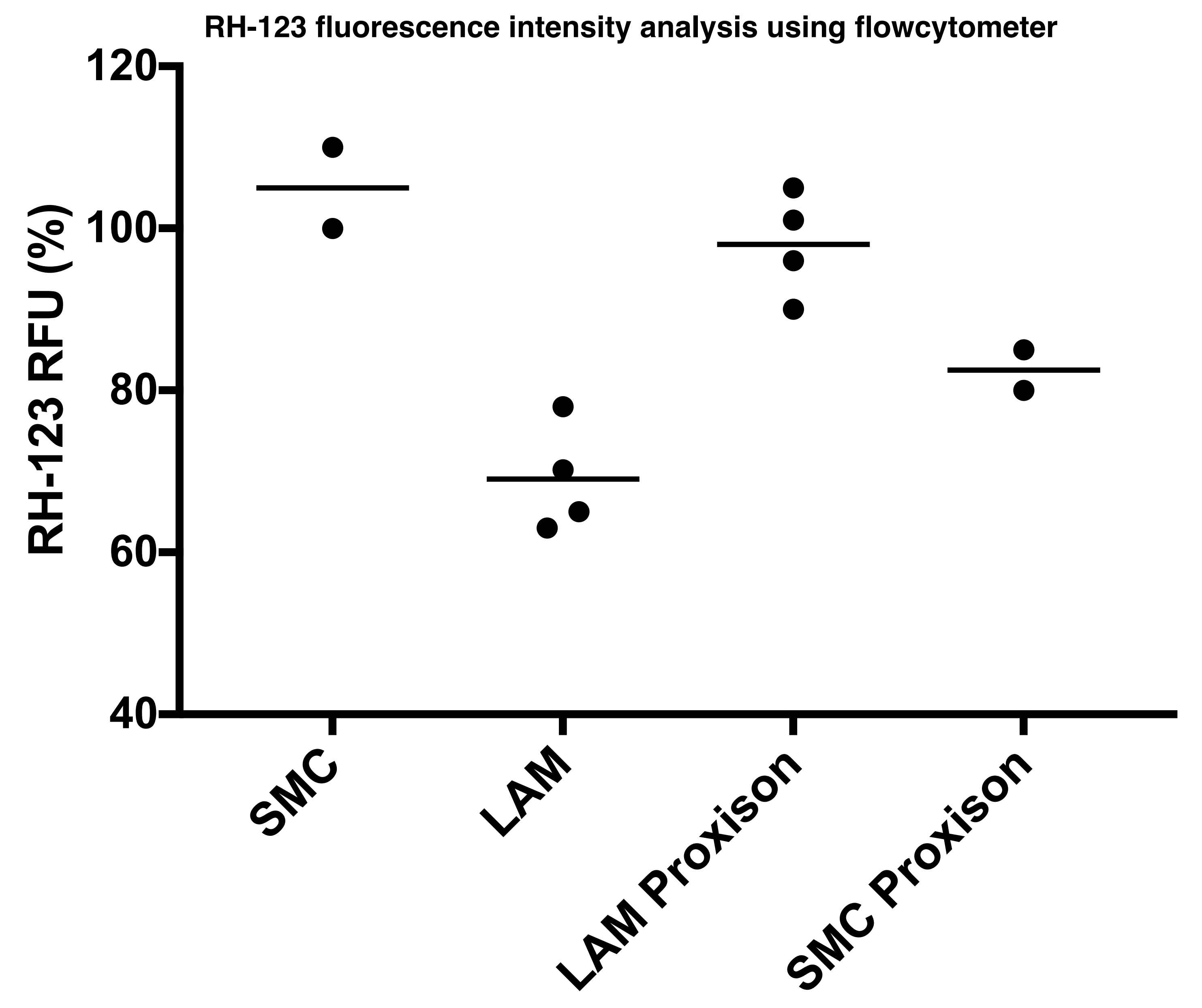

RH-123 fluorescence intensity analysis using flowcytometer
RH-123 RFU (%)
120
100
80
60
40
SMC
LAM
LAM Proxison
SMC Proxison

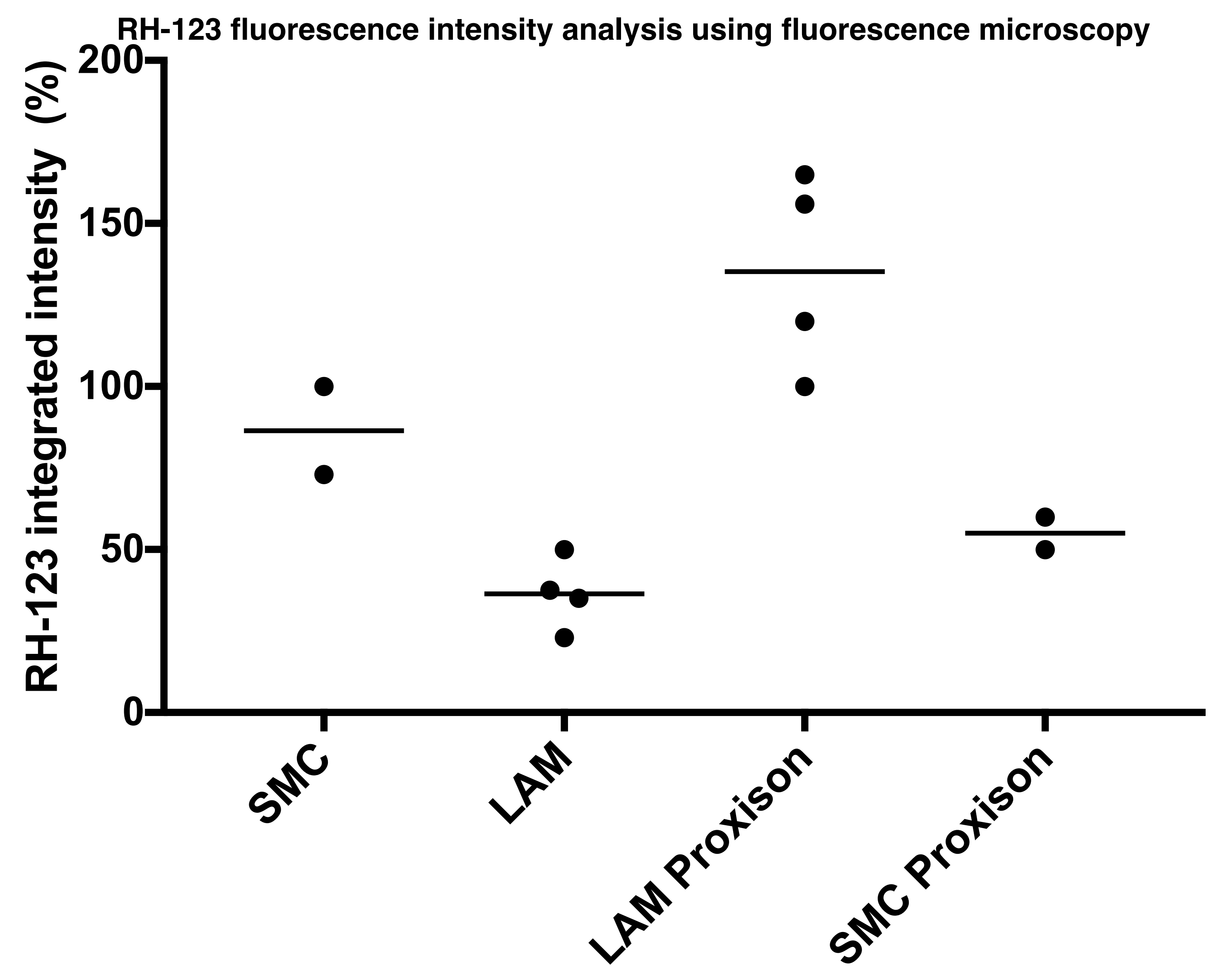
RH-123 fluorescence intensity analysis using fluorescence microscopy
RH-123 integrated intensity (%)
200
150
100
50
0
SMC
LAM
LAM Proxison
SMC Proxison

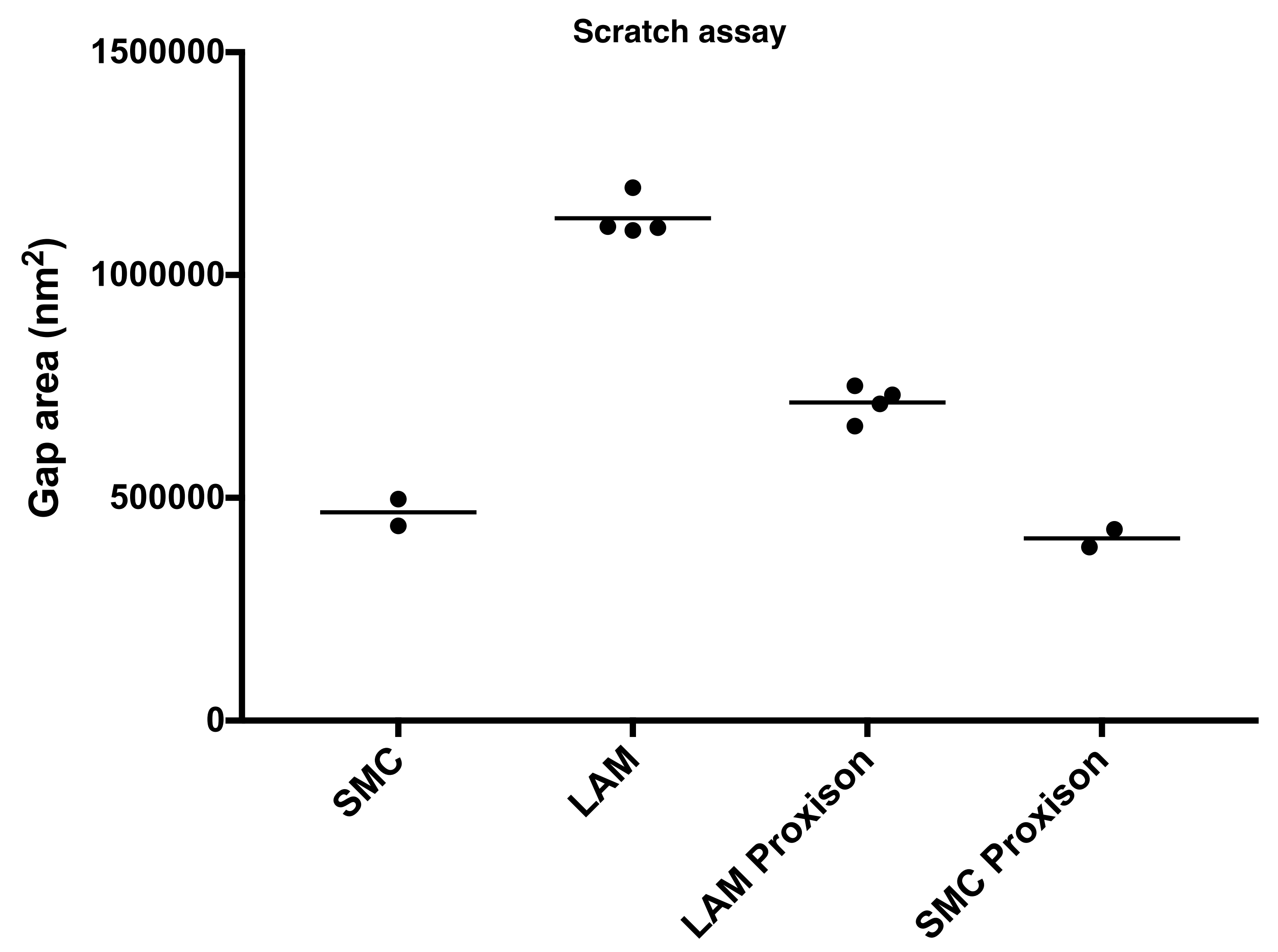
Scratch assay
Gap area (nm2)
1500000
1000000
500000
0
SMC
LAM
LAM Proxison
SMC Proxison

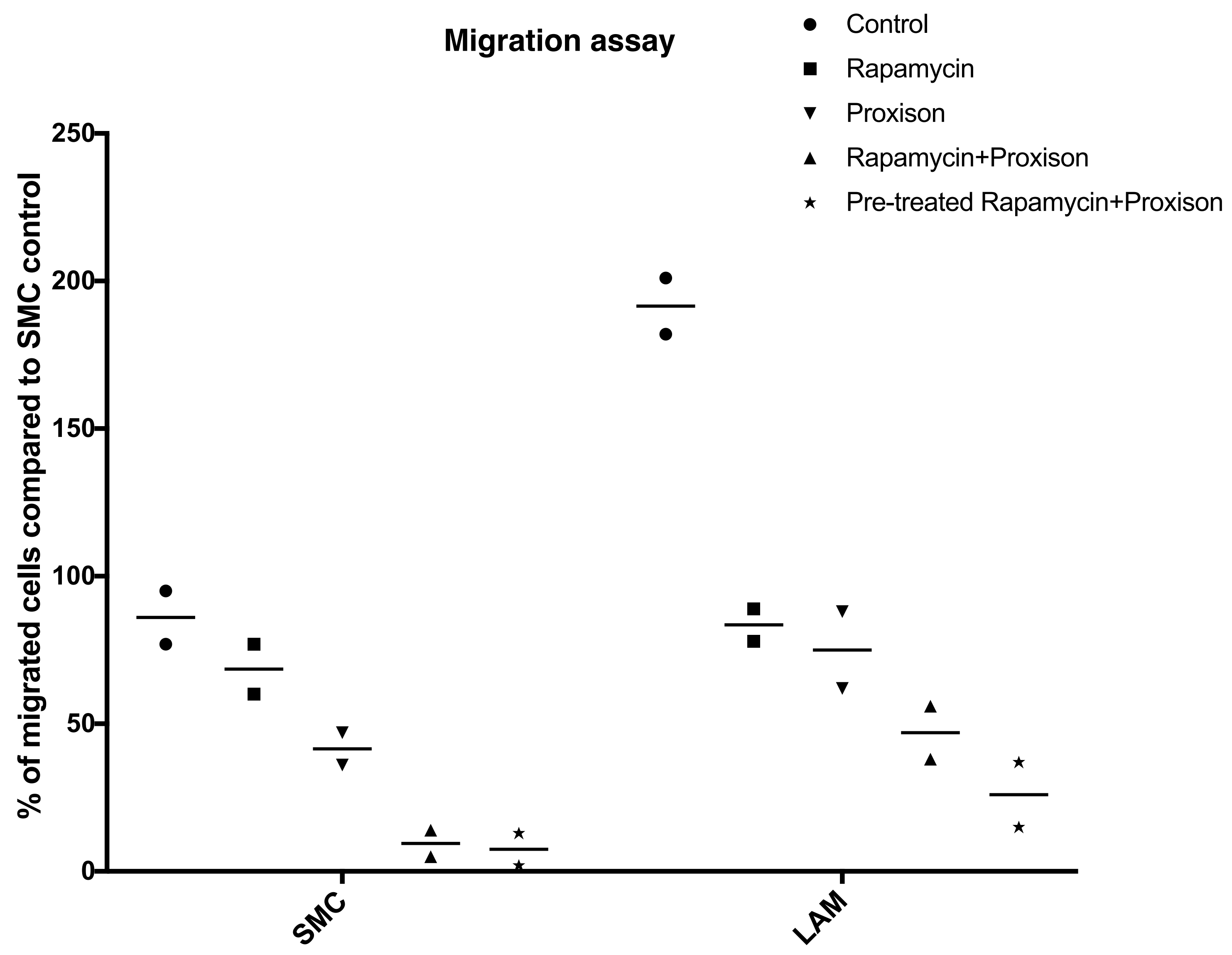
Migration assay
Control
Rapamycin
Proxison
Rapamycin+Proxison
Pre-treated Rapamycin+Proxison
% of migrated cells compared to SMC control
250
200
150
100
50
0
SMC
LAM
